# SUMO Localizes to the Central Element of Synaptonemal Complex and Is Required for the Full Synapsis of Meiotic Chromosomes in Budding Yeast

**DOI:** 10.1371/journal.pgen.1003837

**Published:** 2013-10-03

**Authors:** Karen Voelkel-Meiman, Louis F. Taylor, Pritam Mukherjee, Neil Humphryes, Hideo Tsubouchi, Amy J. MacQueen

**Affiliations:** 1 Department of Molecular Biology and Biochemistry, Wesleyan University, Middletown, Connecticut, United States of America; 2 MRC Genome Damage and Stability Centre, University of Sussex, Brighton, United Kingdom; University of California Davis, United States of America

## Abstract

The synaptonemal complex (SC) is a widely conserved structure that mediates the intimate alignment of homologous chromosomes during meiotic prophase and is required for proper homolog segregation at meiosis I. However, fundamental details of SC architecture and assembly remain poorly understood. The coiled-coil protein, Zip1, is the only component whose arrangement within the mature SC of budding yeast has been extensively characterized. It has been proposed that the Small Ubiquitin-like MOdifier, SUMO, plays a role in SC assembly by linking chromosome axes with Zip1's C termini. The role of SUMO in SC structure has not been directly tested, however, because cells lacking SUMO are inviable. Here, we provide direct evidence for SUMO's function in SC assembly. A meiotic *smt3* reduction-of-function strain displays reduced sporulation, abnormal levels of crossover recombination, and diminished SC assembly. SC structures are nearly absent when induced at later meiotic time points in the *smt3* reduction-of-function background. Using Structured Illumination Microscopy we furthermore determine the position of SUMO within budding yeast SC structure. In contrast to previous models that positioned SUMO near Zip1's C termini, we demonstrate that SUMO lies at the midline of SC central region proximal to Zip1's N termini, within a subdomain called the “central element”. The recently identified SUMOylated SC component, Ecm11, also localizes to the SC central element. Finally, we show that SUMO, Ecm11, and even unSUMOylatable Ecm11 exhibit Zip1-like ongoing incorporation into previously established SCs during meiotic prophase and that the relative abundance of SUMO and Ecm11 correlates with Zip1's abundance within SCs of varying Zip1 content. We discuss a model in which central element proteins are core building blocks that stabilize the architecture of SC near Zip1's N termini, and where SUMOylation may occur subsequent to the incorporation of components like Ecm11 into an SC precursor structure.

## Author Summary

The meiotic cell cycle enables sexually reproducing organisms to generate reproductive cells with half their chromosome complement. Chromosome ploidy is reduced during meiosis by virtue of prior associations established between homologous chromosomes (homologs). Such associations, which are ultimately secured by crossover recombination events, allow homologs to achieve an opposing orientation and segregate from one another at meiosis I. A multimeric protein structure, the synaptonemal complex (SC), mediates the intimate, lengthwise alignment of homologs during meiotic prophase and forms the context in which crossovers mature. The SC's tripartite structure is widely conserved but its composition and architecture remain incompletely understood in any organism. The Small Ubiquitin-like MOdifier (SUMO) localizes to SC in budding yeast. We show that SUMO is required for assembling mature SC and we furthermore demonstrate that SUMO and the recently identified SUMOylated protein, Ecm11, are components of the central element substructure of the budding yeast SC. Our findings suggest that SUMO and Ecm11 are core building blocks of SC, yet our data also suggest that SUMOylation may occur subsequent to Ecm11's incorporation into the SC structure. Finally, our study highlights Structured Illumination as a powerful tool for mapping the fine structure of budding yeast SC.

## Introduction

Chromosomes must form enduring attachments with their homologous partners in order to successfully orient and segregate during the first meiotic division. Such pair-wise chromosomal attachments are ultimately generated by interhomolog crossover recombination events, which occur through the repair of programmed, double-stranded DNA breaks using the homologous partner chromosome [1], [2], [3]. The synaptonemal complex (SC), a multimeric protein structure that normally assembles downstream of initial homology recognition between partner chromosomes, mediates the close, lengthwise apposition of homologous chromosomes (synapsis) during mid-meiotic prophase and is required for a proper number and distribution of interhomolog crossover recombination events [4].

Fundamental details of SC structure and its assembly remain poorly understood. Ultrastructural studies in several different organisms led to the description of at least three substructures that define SC [5], [6]: first, a synapsed pair of chromosomes exhibit two electron dense structures, termed lateral elements, that lie in parallel to one another. Lateral elements correspond to the axial cores of each homolog which organize and maintain cohesion between sister chromatids, and which contain several meiosis-specific components, including the Red1 protein in *Saccharomyces cerevisiae* (budding yeast) [7]. A less electron-dense domain, called the central region, connects lateral elements of aligned homologs along their entire length. In many preparations, two distinct substructures within the SC central region itself are visible: transverse filaments are oriented perpendicular to lateral elements and span the central region, while a structure called the central element is oriented in parallel to lateral elements at the midline of the SC central region.

Proteins that localize to SC have been identified in several different organisms. However, despite an overall conservation of SC structure among different organisms, the lack of sequence homology between SC proteins belonging to different species means that substantial cytological and genetic characterization must be gathered for each identified SC protein, in order to determine its position within the higher order SC structure and its role in SC assembly. One conserved molecular aspect of SC among organisms is found in the secondary structure of transverse filament proteins; these components contain extensive coiled-coil motif and are predicted to form parallel homodimers [4]. Dimers of the *S. cerevisiae* transverse filament protein, Zip1, are predicted to generate a ∼50–80 nm rod with globular ends [8], [9]. In elegant immuno-electron microscopy experiments using Zip1 domain-specific antibodies, Dong and Roeder (2000) demonstrated that two Zip1 coiled-coil units (either dimers or tetramers) span the width of the SC, with their N termini interacting at the center of the structure and their C termini oriented toward chromosome axes [10]. Similar immuno-EM localization experiments have supported this overall structural orientation for transverse filament proteins in both *Drosophila* (C(3)G) and mammals (Syp1) [11], [12], [13].

Additional proteins that localize to SC but do not contain coiled-coil have been identified in yeast and other organisms [4], [11], [14], [15], [16], [17], [18], [19], [20], [21]. For instance, four proteins (SYCE1, SYCE2, SYCE3, TEX12) have been found to localize to the central element structure at the midline of the SC in mouse [15], [16], [18], [22], [23]. Loss-of-function of any of these proteins results in abolished or fragmented SC assembly but, in some cases, no defect in the localization of transverse filament proteins to chromosomes *per se*, suggesting that an interaction between central element proteins and transverse filaments is essential to drive the multimeric assembly of mature SC. Consistent with this idea, Jeffress et al. (2007) found that the N terminal region of the *Drosophila* transverse filament protein, C(3)G, is dispensable for the loading of C(3)G onto meiotic chromosomes but is critical for SC assembly [24]. Proteins defining the central element in budding yeast have not been identified to date.

The Small Ubiquitin-like MOdifier protein, SUMO, robustly localizes coincident with Zip1 and in a Zip1-dependent manner at the interface of length-wise aligned, synapsed chromosomes in budding yeast prophase nuclei [19] and thus may serve a structural or regulatory role in SC assembly. A role for SUMO in SC formation was suggested by the observation that a tagged version of the E2 SUMO ligase, Ubc9, results in disrupted SC assembly [19]. Moreover, several reports have suggested that SUMO or SUMO chains link Zip1's C termini to chromosome axes through an interaction between SUMOylated Red1 (a meiotic chromosomal core component) and Zip1's C terminal SUMO Interacting Motif (SIM) [25], [26], [27], [28], although one of these studies provided evidence that strains expressing an unSUMOylatable *red1* allele did not have SC assembly defects [27]. A direct functional role for SUMO in SC assembly or structure has not been rigorously demonstrated.

Here we directly investigate the role of SUMO in budding yeast SC assembly. We find that SUMO and Zip1 are dependent on one another for full synapsis, while SUMO is likely dispensable for Zip1 polycomplex formation. Consistent with our SUMO loss-of-function analysis, the SUMOylated SC component Ecm11 was recently shown to be required for SC assembly [29], [30], [31]. We furthermore report that like Zip1, SUMO and Ecm11-MYC continuously incorporate into full-length SC during meiotic prophase. Finally, using superresolution microscopy we demonstrate that SUMO and the SUMOylated Ecm11 protein predominantly localize to a discrete zone corresponding to the central element of the SC. This latter observation challenges the idea that SUMO interacts with Zip1's C terminal domain within assembled SC, and adds to the accumulating evidence that central element proteins play a conserved role in facilitating the assembly and/or maintenance of the mature SC structure.

## Results

### SUMO-Diminished Meiotic Cells Progress to Late Prophase but Exhibit a *PCH2*-Dependent Decline in Sporulation Efficiency

**Figure 1:**
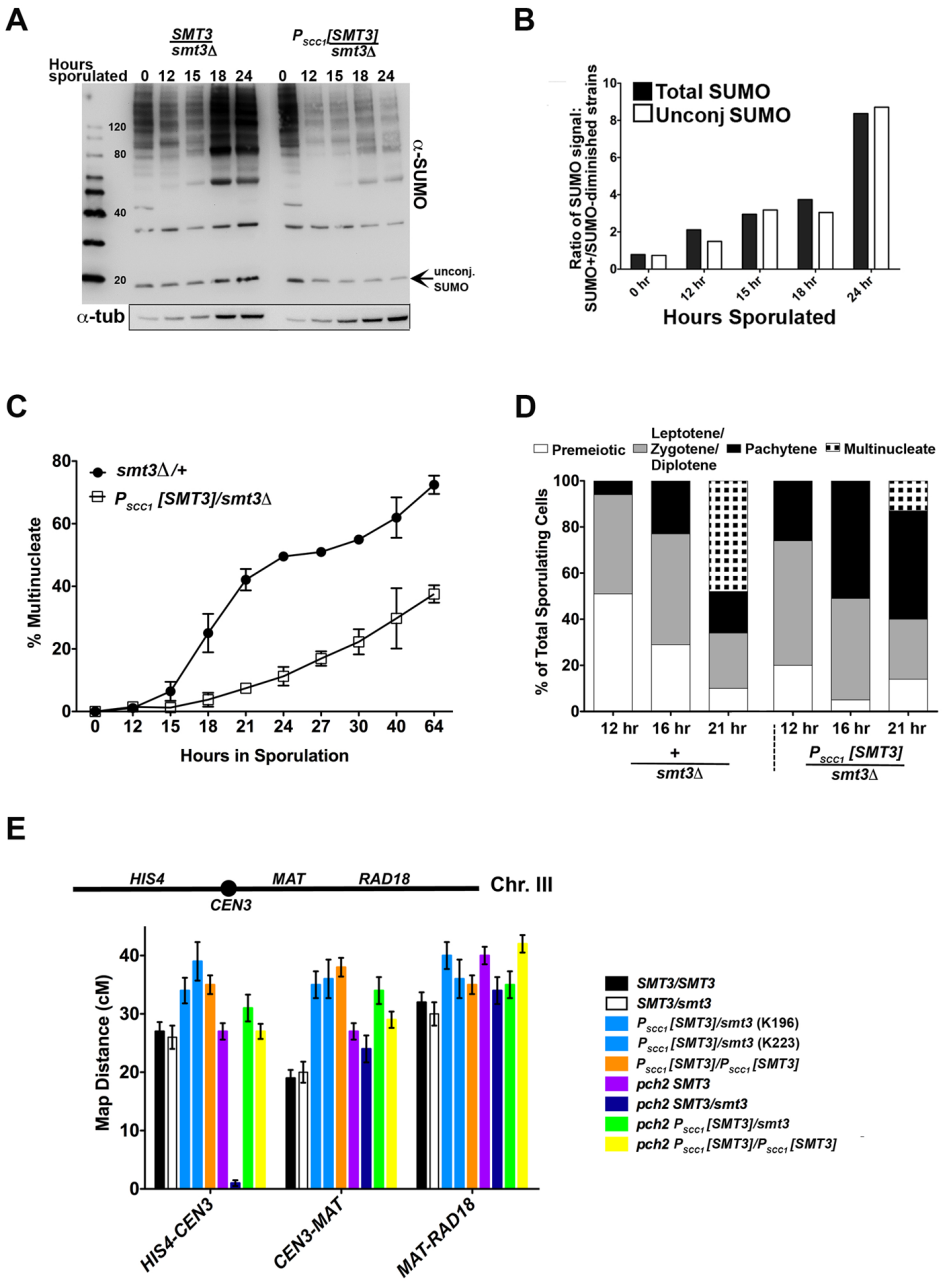
SUMO-diminished strains exhibit reduced sporulation and elevated crossover levels. (A) Western blot detecting SUMO in lysates from control (*smt3Δ/+*, LFT36) and SUMO-diminished (*P_SCC1_[SMT3]/smt3Δ*, LFT46) cells at 0, 12, 15 18 and 24 hours of sporulation. Bar graph in (B) gives the ratio of conjugated SUMO protein levels in LFT36 over conjugated SUMO protein levels in LFT46 (solid bars), as well as the ratio of unconjugated (free) SUMO levels in LFT36 over LFT46 (open bars) at each time point. (C) *smt3Δ/+* (LFT36, closed circles) and *P_SCC1_[SMT3]/smt3Δ* (LFT46, open squares) cells were sporulated and then assessed for the formation of multinucleate products over a time course (time points indicated on x axis). (For each strain at any given time point, the average between two independent experiments is indicated (in total, >800 nuclei were scored for each timepoint.) In (D), control and *P_SCC1_[SMT3]/smt3Δ* cells were sporulated and surface spread on glass slides at the time points indicated in order to assess whether SUMO-diminished strains enter into and progress through meiotic prophase efficiently. The fraction of nuclei that had entered meiosis at each time point was recorded based on Red1 staining (not shown), while the fraction of nuclei that had reached the pachytene stage of meiotic prophase (or post-MI) were recorded based on the morphology of the DAPI-stained nucleus. Meiotic (Red1-positive) nuclei without pachytene morphology were grouped as the “Leptotene/Zygotene” (pre-pachytene) or “Diplotene” (post-pachytene) category. (E) Cartoon above graph indicates the genetic intervals used to calculate map distances on chromosome III. *CEN3* is marked with an adjacent hygromycin resistance cassette and the *RAD18* locus is marked with *ADE2*. Bar graph displays map distances (cM) for the three intervals (x axis) measured in control and SUMO-diminished strains. Error bars represent the standard error of map distance. See Table 2 for precise values and for map distances additionally calculated using tetrad analysis.

A diploid strain (LFT46) was created in which *SMT3* (encoding the SUMO precursor protein) on one chromosome is under the control of the mitosis-specific *MCD1/SCC1* promoter [32], [33], [34] while the other *SMT3* copy is deleted. The level of SUMO signal in lysates from *P_SCC1_[SMT3]/smt3Δ* cells was reduced, relative to *+/smt3Δ* heterozygous control (LFT36) cells, at 12, 15, 18 and 24 hours during a meiotic time course (Figure 1A, 1B). The difference in SUMO levels between control and experimental strains was progressively greater at later stages of the time course; overall levels of both SUMO-conjugated proteins and unconjugated (free) SUMO were eight fold lower in *P_SCC1_[SMT3]/smt3Δ* cells relative to control cells at 24 hours of sporulation (Figure 1A, B).

Spore formation was reduced in *P_SCC1_[SMT3]/smt3Δ* (“SUMO-diminished”) cells, as compared to control (*smt3Δ/+* heterozygous) cells (13% versus 54%, Table 1). DAPI-stained whole cells that had been paraformaldehyde-fixed at various times during a sporulation time course were also assessed in order to measure the fraction of cells with multinucleate products (including encapsulated spores) at each time point (Figure 1C). Consistent with the defect in spore production, *P_SCC1_[SMT3]/smt3Δ* cultures exhibited a dramatic reduction in multinucleates at each time point, as compared to the control (*SMT3/smt3Δ*) strain.

**Table 1:** Sporulation efficiency and spore viability in SUMO-diminished strains.

			Distribution of tetrad types					
Strain	Sporulation efficiency % (n)	Tetrads dissected	4-sv	3-sv	2-sv	1-sv	0-sv	Spore viability %
*SMT3/SMT3* (PM89, K189)	54 (3360)	307	268	28	9	1	1	96
*SMT3/smt3* (LFT36, K197)	54 (3002)	313	1*	4*	280	25	3	48
*P_SCC1_[SMT3]/smt3* (LFT46, K196, K223)	13 (6103)	537	6*	1*	435	74	21	45
*pch2/pch2 SMT3/SMT3* (K167)	54 (2071)	285	212	54	14	4	1	91
*pch2/pch2 SMT3/smt3* (K325)	58 (2034)#	410	0	6	320	69	15	44
*pch2/pch2 P_SCC1_[SMT3]/smt3* (LFT85, K198)	60 (5012)	388	2*	1*	296	72	17	43
*P_SCC1_[SMT3]/P_SCC1_[SMT3]* (K188)	44 (2003)	255	192	35	24	3	1	91
*pch2/pch2 P_SCC1_[SMT3]/P_SCC1_[SMT3]* (K191)	62 (2031)	310	225	64	14	4	3	91

Viability of spores produced by diploid *SMT3* homozygotes, *smt3*Δ/+ heterozygotes, *P_SCC1_[SMT3]/P_SCC1_[SMT3]* homozygotes, or *P_SCC1_[SMT3]/smt3*Δ transheterozygotes. The far right column shows the overall spore viability of each strain. Note that as *smt3* haploid cells are inviable, 50% overall spore viability is the maximum expected for strains carrying an *smt3* deletion allele. Displayed in each “Distribution of tetrad types” column is the frequency of tetrads containing four viable spores (4-sv), three viable spores (3-sv), two viable spores (2-sv), one viable spore (1-sv) or no viable spores (0-sv). Full strain genotypes are listed in Table S1.

# Sporulation efficiency in this case was performed on K199, which has the same genotype as K325 but was generated independently.

* Cases of 3- or 4-spore viables derived from strains carrying *smt3::natMX* (K196, K197, K224) arose from non-mendelian segregation events; each colony from dissected ascii displayed sensitivity to nourseothricin, indicating an absence of the *smt3::natMX* knockout allele.

The sporulation defect of SUMO-diminished meiotic cells is at least partially explained by a decreased capacity to transition from late prophase to the first meiotic division. We assessed the capacity of cells to progress into and through earlier meiotic prophase stages by assessing DNA morphology and the presence of the meiotic chromosome axis protein, Red1, on chromosome spreads of sporulating cells. We assessed the fraction of surface-spread nuclei from control (*SMT3+/smt3Δ*) or *P_SCC1_[SMT3]/smt3Δ* cells that had progressed into meiosis (exhibited Red1 staining) at either 12, 16 or 21 hours of sporulation. Based on the morphology of the DAPI-stained nucleus, we could moreover distinguish those nuclei that had progressed to the pachytene stage of meiotic prophase (when homologous chromosomes are lengthwise-aligned and synapsed), and those that had progressed beyond the second meiotic division and had formed encapsulated haploid nuclei. At 12 and 16 hours, we observed an equal or greater fraction of Red1-positive and pachytene stage nuclei in *P_SCC1_[SMT3]/smt3Δ* sporulating cells as compared to control cells (Figure 1D, data not shown), indicating that *P_SCC1_[SMT3]/smt3Δ* cells are competent to enter the meiotic program and progress to the pachytene stage without delay. At the 21 hour time point, a deficit in the number of multinucleates was again observed for SUMO-diminished cells.

We also stained surface-spread nuclei from an independent sporulation time course with both Red1 (not shown) and alpha-tubulin antibody (Figure S1), in order to directly assess progression through the first and second meiotic divisions in control and *P_SCC1_[SMT3]/smt3Δ* cells. The diplotene stage can be distinguished from leptotene/zygotene (pre-pachytene meiotic stages) based on the presence of a duplicated spindle pole body, revealed by alpha-tubulin staining [35], [36], [37]. We analyzed the fraction of Red1-positive nuclei that were either in pre-pachytene or pachytene, diplotene, MI or MII stages in wild type or *P_SCC1_[SMT3]/smt3Δ* cells at 15, 20 or 24 hours of sporulation. Encapsulated spores were not included in this analysis. We found a dramatic reduction in the number of meiotic nuclei containing MI or MII spindles in *P_SCC1_[SMT3]/smt3Δ* cells (Figure S1B). Instead, the vast majority of meiotic nuclei analyzed from SUMO-diminished cells appeared to be in pre-pachytene or pachytene stages.

Taken together, these analyses indicate that *P_SCC1_[SMT3]/smt3Δ* cells efficiently progress to the pachytene stage of meiosis, but are delayed in progressing from the pachytene stage of meiotic prophase into meiosis I.

Pch2 enforces a late prophase meiotic checkpoint that is triggered by defects in recombination and synapsis [38], [39], [40], [41], [42]. For example, *zip1* mutants fail to build SC and exhibit reduced levels of crossover recombination and are delayed in progressing from late prophase into meiosis I [8]. However, *pch2 zip1* double mutants progress through the meiotic divisions and generate spores at wild-type levels [40]. In order to ask whether *P_SCC1_[SMT3]/smt3Δ* cells trigger a Pch2-dependent checkpoint, we assessed sporulation efficiency of a *P_SCC1_[SMT3]/smt3 pch2*Δ/*pch2*Δ strain (LFT85). Indeed, deletion of *PCH2* improved spore formation in *P_SCC1_[SMT3]/smt3Δ* cells (60% in *pch2* versus 13% in *PCH2+*, Table 1). This phenotype may reflect a role for SUMO in either SC assembly or in meiotic recombination.

The overall viability of spores produced by *PCH2+* or *pch2 Δ P_SCC1_[SMT3]/smt3Δ* strains was not significantly different from control cells (Table 1). However, in (*PCH2+*) *P_SCC1_[SMT3]/smt3Δ* versus *smt3Δ/+* strains, the fraction of 2-spore viable tetrad types was significantly reduced (two sided P value = 0.0013, Fisher's Exact test), and the fraction of 0- and 1-spore viables among the total spores was significantly increased (two-sided P value = 0.0004 for the 0- and 1-spore viable classes combined, Fisher's Exact test).

### SUMO-Diminished Strains Exhibit Increased Crossover Recombination on Chromosome III

Interhomolog crossover recombination was assessed using three intervals on chromosome III in *SMT3+*, *+/smt3Δ*, and *P_SCC1_[SMT3]/smt3Δ* strains as well as *P_SCC1_[SMT3]/P_SCC1_[SMT3]* homozygotes. As only 2-spore viable products result from most meioses in strains carrying an *SMT3* deletion, we calculated crossover recombination frequency by measuring the fraction of colonies carrying a recombinant chromosome among the total population of viable colonies (Figure 1E, Table 2). Map distances for each interval on chromosome III were 1.2–1.5-fold increased in both *P_SCC1_[SMT3]/smt3Δ* and *P_SCC1_[SMT3]/P_SCC1_[SMT3]* backgrounds relative to *SMT3+* and *+/smt3Δ* control strains.

**Table 2:** Chromosome III map distances in SUMO-diminished strains.

		100×Recombinant chromosome III Total chromosome IIIs			
Strain	Spores	*HIS4-CEN3* (S.E)	*CEN3-MAT* (S.E)	*MAT-RAD18* (S.E)	Total III
*SMT3/SMT3* (K189)	756	27 (1.6)	19 (1.4)	32 (1.7)	78
*SMT3/smt3* (K197)	480	26 (2.0)	20 (1.8)	30 (2.0)	76
*P_scc1_[SMT3]/smt3* (K196)	432	34 (2.2)	35 (2.3)	40 (2.3)	109
*P_scc1_[SMT3]/smt3* (K223)	218	39 (3.3)	36 (3.3)	36 (3.3)	111
*P_scc1_[SMT3]/P_scc1_[SMT3]* (K188)	921	35 (1.6)	38 (1.6)	35 (1.6)	108
*pch2/pch2 SMT3/SMT3* (K167)	1015	27 (1.4)	27 (1.4)	40 (1.5)	94
*pch2/pch2 SMT3/smt3* (K325)	467	25 (2)	23 (1.9)	41 (2.3)	89
*pch2/pch2 P_scc1_[SMT3]/smt3* (K198)	416	31 (2.3)	34 (2.3)	35 (2.3)	100
*pch2/pch2 P_scc1_[SMT3]/P_scc1_[SMT3]* (K191)	1113	27 (1.3)	29 (1.4)	42 (1.5)	98

		cM [100(6N+T)/2(P+N+T)]			
Strain	Tetrads	*HIS4-CEN3* (S.E.)	*CEN3-MAT* (S.E.)	*MAT-RAD18* (S.E.)	Total III
*SMT3/SMT3* (K189)	175	28 (.025)	18 (.018)	38 (.037)	84
*P_scc1_[SMT3]/P_scc1_[SMT3]* (K188)	192	34 (.038)	29 (.031)	41 (.030)	104
*pch2/pch2 SMT3/SMT3* (K167)	205	34 (.040)	31 (.030)	52 (.049)	117
*pch2/pch2 P_scc1_[SMT3]/P_scc1_[SMT3]* (K191)	218	29 (.029)	30 (.022)	54 (.045)	113

		Interference			
Strain	TT	NPD^obs^	NPD^exp^	NPD Ratio	Prob.
***SMT3/SMT3*** *HIS4-CEN3*	86	1	8.6	0.1	**0.02**
**(K189)** *CEN3-MAT*	60	0	3	ND	ND
*MAT-RAD18*	102	4	14.5	0.3	**0.02**
***P_scc1_[SMT3]/P_scc1_[SMT3]*** *HIS4-CEN3*	95	5	9.6	0.5	0.29
**(K188)** *CEN3-MAT*	91	3	8.5	0.4	0.14
*MAT-RAD18*	130	3	37	0.1	**<.0001**
***pch2/pch2 SMT3/SMT3*** *HIS4-CEN3*	92	7	7.8	0.9	1
**(K167)** *CEN3-MAT*	105	3	11.3	0.3	**0.05**
*MAT-RAD18*	128	12	22	0.5	0.11
***pch2/pch2*** *HIS4-CEN3*	101	3	9.2	0.3	0.14
***P_scc1_[SMT3]/P_scc1_[SMT3]*** ** (K191)** *CEN3-MAT*	116	3	13.5	0.2	**0.01**
*MAT-RAD18*	147	12	41	0.3	**<.0001**

Strains carrying an *smt3* deletion allele give predominantly 2-spore viables, thus random spore analysis was used to calculate map distances and standard errors between three intervals on chromosome III in all SUMO-diminished and control backgrounds (see Methods). Cen3 was marked using a hygromycin resistance cassette and *RAD18* was marked using the *ADE2* gene. Standard error (S.E.) values were calculated according to the formula: 100(√(r/t)(1-r/t)/t), where *r* = number of recombinant colonies and *t* = total number of colonies counted. Additionally (middle section of the table), map distances were calculated using tetrad analysis as per Perkins [56]. Tetrad analysis to generate genetic distances and standard error (S.E.) values using tetrad data were calculated using the Stahl lab online tools: http://molbio.uoregon.edu/~fstahl/. For 175 tetrads in K189 cells, two (3∶1) segregation events were evident at the *HIS4* locus, one event occurred at the *MAT* locus and four events (all 3∶1 *ADE2+:ade2)* occurred at *RAD18*. For 192 tetrads from K188 cells, five (3∶1) segregation events were evident at *HIS4*, two events occurred at *MAT*, and nine events (seven (3∶1) and two (1∶3) *ADE2+:ade2* events) occurred at *RAD18*. Interference values (lower section of table) were calculated using tetrad analysis data. Interference was measured by calculating the ratio of Non Parental Ditype tetrads (NPDs) observed over the NPDs expected (values less than one reflect positive interference). NPDs expected for each interval were calculated using the fraction of tetratypes (TT) observed in each dataset according to the formula: NPD^exp^ = 1/2(1-*f*TT-[1-*3fTT*/2]^2/3^ (where *f*TT = fraction of tetratypes) [59]. Numbers in the “Prob.” column represent the two-sided P value (Fisher's Exact Test) that describes the probability that the difference between observed and expected NPD proportions are due to chance. Prob. values in bold indicate statistical significance.

We additionally measured map distances in *SMT3+* and *P_SCC1_[SMT3]/P_SCC1_[SMT3]* strains using standard tetrad analysis (Table 2, middle section). This assessment also found an increase in interhomolog crossover recombination for *P_SCC1_[SMT3]/P_SCC1_[SMT3]* relative to *SMT3+* strains.

We assessed interhomolog crossover recombination in *SMT3+* and SUMO-diminished strains homozygous for a *pch2* null allele, the mutation that suppresses the sporulation defect associated with SUMO-diminishment (see above). All *pch2* mutant strains (controls and SUMO-diminished) exhibited elevated levels of interhomolog crossover events, relative to *PCH2+* strains, in the three chromosome III intervals assessed. In the absence of Pch2, *SMT3+* and SUMO-diminished strains exhibited less disparity in genetic distances on III, and in cases where a difference in map distance is observed there lacks a consistent pattern in the direction of recombination levels relative to controls.

Notably, tetrad analysis on *pch2 SMT3*+ versus *pch2 P_SCC1_[SMT3]/P_SCC1_[SMT3]* strains (Table 2, middle section) showed no significant alteration in map distances on any of the three chromosome III intervals, whereas a significant increase in map distance was found for all three intervals on III for *PCH2+ P_SCC1_[SMT3]/P_SCC1_[SMT3]*, relative to *SMT3+* strains.

Crossover recombination events during meiosis typically exhibit positive interference, meaning their distribution is such that no two crossovers occur close together. The SC is involved in achieving crossover interference, as the residual crossovers that occur in synapsis-defective mutants do not exhibit positive interference [43]. We analyzed crossover interference in control and *P_SCC1_[SMT3]* homozygotes using our tetrad analysis data (Table 2, lower section; see legend for methods). Our analysis of this dataset revealed that the interhomolog crossovers in all strains tested exhibit interference, although the statistical significance of interference values was weaker in some intervals (reflecting a higher probability that crossovers exhibit an interfering distribution due to chance) for *P_SCC1_[SMT3]* homozygotes and both *pch2* strains, relative to the *PCH2+ SMT3+* control.

Taken together, our recombination data indicate that SUMO-diminishment leads to an increase in interhomolog crossover recombination events, and further raise the possibility that Pch2 and SUMO may negatively regulate interhomolog crossovers in a similar pathway.

### SUMO Is Required for Full SC Assembly

If they comprise equally fundamental structural components of SC central region, then SUMO and Zip1 may be mutually dependent on one another for their function in SC assembly. In order to directly ask whether SC assembly relies on SUMO, we analyzed Zip1 distribution on chromosomes in nuclei from *P_SCC1_[SMT3]/smt3Δ* (LFT47) or control (*smt3Δ/+*, LFT37) cells after ∼24 hours of sporulation. In this analysis, both experimental and control strains were homozygous for a gene encoding the tagged version of Zip1, *ZIP1-GFP* [44], and for an *ndt80* null allele. *NDT80* activity is required for progression beyond the pachytene stage, when chromosomes exhibit full-length SC. Thus, after 24 hours of sporulation most nuclei from *ndt80* homozygous strains have progressed to the pachytene stage.

**Figure 2:**
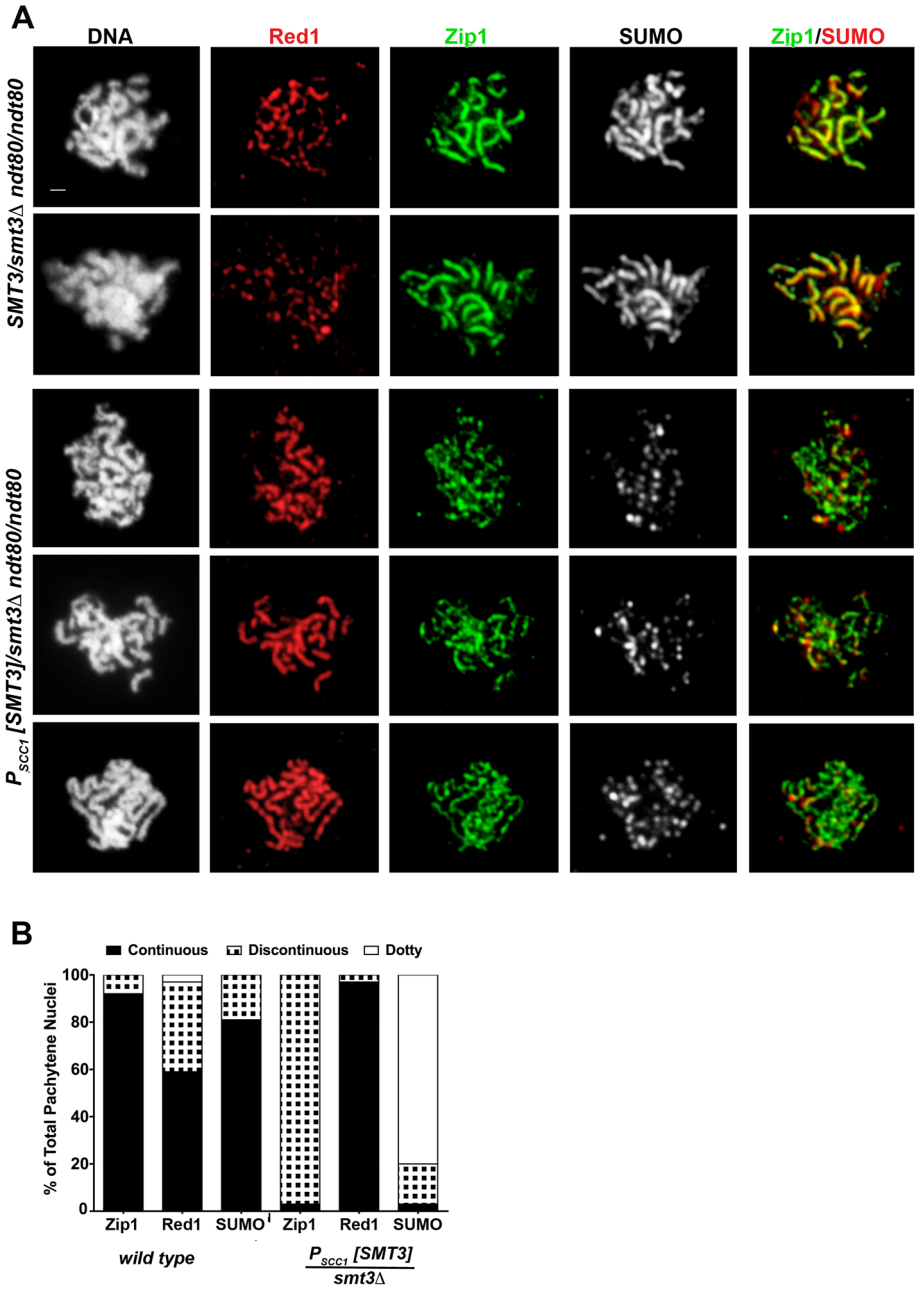
SC appears discontinuous in SUMO-diminished pachytene nuclei. (A) Shown are surface-spread meiotic nuclei from *smt3Δ/+* (LFT37) and *P_SCC1_[SMT3]/smt3Δ* (LFT47) *ndt80/ndt80* strains that had been sporulated for 24 hours. DAPI (white, first column) and antibodies to Red1 (red), Zip1 (green), SUMO (white, 3rd column; red, 4th column) were used to stain nuclei and assess SC formation. Scale, 1 µm. (B) displays the percentage of nuclei (n>50 per column) from control or SUMO-diminished strains that exhibit either continuous (black), discontinuous (black/white hatched), or dotty pattern of Zip1, Red1, or SUMO staining.

As has been previously reported, surface-spread pachytene stage nuclei from control cells exhibit extensive linear stretches of Zip1-GFP coincident with SUMO at the interface of synapsed homologous chromosomes, while the meiotic chromosome axis protein, Red1, often (∼40% of the time in this analysis, n = 50) exhibits a more discontinuous staining pattern along the same interface [7], [19], [45] (Figure 2). Nuclei from *P_SCC1_[SMT3]/smt3Δ* cells that had been sporulating for ∼24 hours typically exhibited foci or faint short stretches of SUMO on pachytene chromosomes (Figure 2A), indicating that SUMO is indeed diminished in these meiotic cells. Zip1 linear stretches appeared more abundantly than SUMO linear stretches on chromosome spreads from *P_SCC1_[SMT3]/smt3Δ* cells, but the linear Zip1 we observed appeared abnormally discontinuous and narrow. Furthermore, Red1 appeared continuous along chromosome axes more frequently than in control nuclei (Figure 2B), reminiscent of the phenotype exhibited by the *zip1* null mutant [7].

The abnormal Zip1 and Red1 staining patterns exhibited by *P_SCC1_[SMT3]/smt3Δ* nuclei suggest a role for SUMO in SC assembly or maintenance. However, since the extent of SUMO-depletion in *P_SCC1_[SMT3]/smt3Δ* cells depends upon the turnover of SUMO and SUMO-conjugated products formed during vegetative growth (when *SMT3* is expressed in this background), the degree of SUMO-diminishment may vary depending on the timing of progression of individual cells through meiosis. Thus, the discontinuous Zip1 phenotype we observed on pachytene chromosomes in SUMO-diminished meiotic cells might reflect a mild phenotype caused by a partial reduction in SUMO during meiotic prophase.

**Figure 3:**
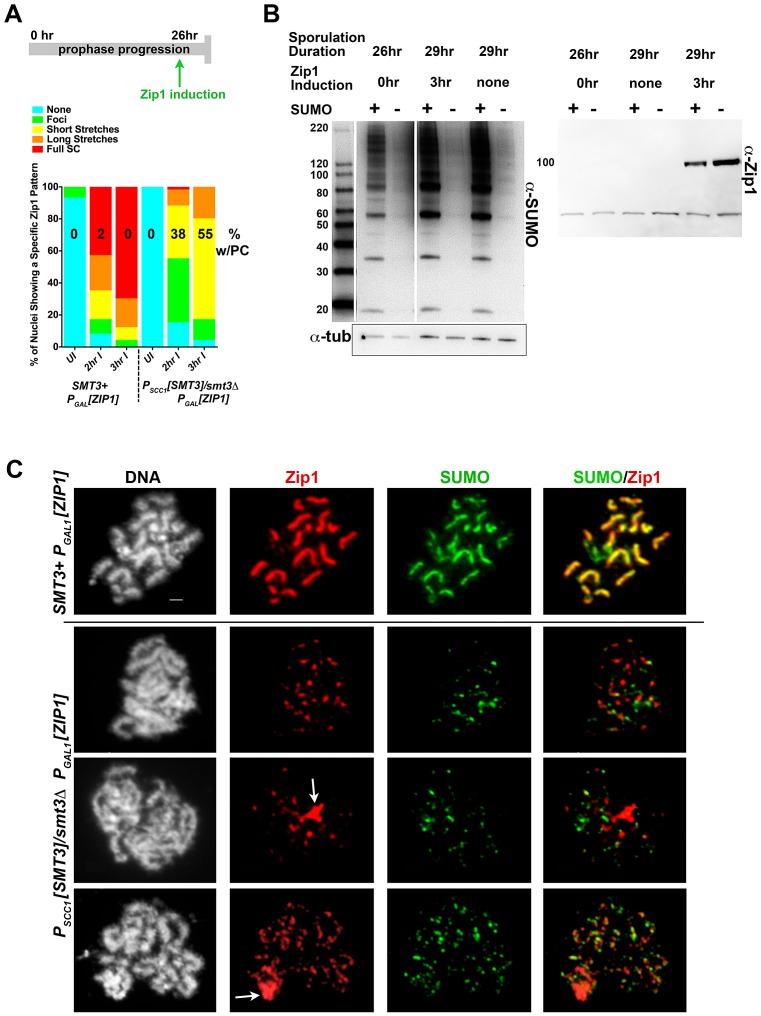
Induced Zip1 assembles full SC stretches in a SUMO-dependent manner. Cartoon in (A) depicts the Zip1 induction experiment conducted, as described ([46], this work) using LFT51 (control) and LFT65 (sumo-diminished) strains. Bar graph indicates the extent of SC assembly (Zip1 staining) on chromosomes at each time point following induction (n>50 for each time point). Number on each column indicates the percentage of each set of nuclei that exhibit a polycomplex (Zip1 aggregate) structure. Western blots in (B) show SUMO levels in cellular lysates from the sporulated cells in this experiment (left blot; SUMO signal is 2–8 fold reduced in SUMO-diminished as compared to *SMT3+* cells) and Zip1 levels in uninduced and induced strains (right blot; Zip1 runs at ∼100 kD, whereas the lower band is nonspecific). (C) Examples of meiotic surface-spread chromosomes from each strain after induction of *ZIP1* expression. Chromosome spreads have been labeled with DAPI (DNA, white), antibodies to Zip1 (red) and SUMO (green). Arrows indicate polycomplex structures. Scale, 1 µm.

In order to maximally diminish SUMO prior to assessing the consequences for SC assembly, we next utilized an inducible Zip1 system. As we previously reported, meiotic chromosomes that have been held at a late prophase arrest in *ndt80* mutant cells are capable of *de novo* SC assembly after *ZIP1* expression is induced, even after 26 hours of sporulation in the absence of *ZIP1* [46]. We used this inducible *ZIP1* system to assess whether meiotic cells, held in sporulation media for 26 hours with diminished *SMT3* expression, are capable of assembling SC *de novo* on chromosomes. SUMO levels in SUMO-diminished (LFT65) lysates from 26 hour, sporulating cultures of our Zip1-induction strains showed up to an eight fold reduction in SUMO conjugates, as compared to the analogous *SMT3+* strain (Figure 3B).

As previously observed, *ndt80* homozygous cells carrying *ZIP1* under the control of a ß-estradiol-inducible *P_GAL1_* system exhibit a *zip1*-like meiotic chromosome phenotype at 26 hours of sporulation in the absence of ß-estradiol [46]. Little Zip1 was detected and SUMO antibodies labeled only foci on chromosome spreads from uninduced cultures of both control and SUMO-diminished genotypes (data not shown).

Control and SUMO-diminished *ndt80* cells carrying inducible *ZIP1* were sporulated for 26 hours and then exposed to ß-estradiol in order to induce *ZIP1* expression. Two hours after induction, surface-spread chromosomes from many control cells (LFT51) exhibited extensive Zip1 with coincident SUMO linear assemblies (Figure 3A, C). In contrast, Zip1 was dramatically reduced on meiotic chromosomes from *P_SCC1_[SMT3]/smt3Δ* cells carrying the *ZIP1* inducible system (LFT65). Even after 3 hours of *ZIP1* induction, most SUMO-diminished meiotic nuclei exhibited only Zip1 foci, or short Zip1 linear stretches that were often unassociated with a SUMO signal (Figure 3A,C). We assessed the organization of Zip1 within these short Zip1 stretches that were sometimes observed in SUMO-diminished nuclei with induced Zip1 and found that they do not reflect normal SC structure; Structured Illumination microscopy (see below, Figure S2) indicated that, unlike control cells where antibodies targeting the C termini of Zip1 often appear as parallel tracts, Zip1-C antibodies decorate chromosomes only as foci or short, single tracts on chromosomes in *P_SCC1_[SMT3]/smt3Δ* cells carrying the *ZIP1* inducible system (Figure S2). We conclude that Zip1 can associate with chromosomes but is not capable of forming mature SC in the vast majority of SUMO-diminished cells with induced Zip1.

### Zip1 Polycomplex Forms in *spo11* Mutants with Diminished SUMO

Zip1 aggregates, resembling the polycomplexes that are a hallmark of strains with a defect in synapsis, were frequently observed in *P_SCC1_[SMT3]/smt3Δ* cells carrying the *ZIP1* inducible system but rarely observed in control nuclei (55% versus <2%, respectively; Figure 3A, C). Interestingly, SUMO was typically undetectable in such Zip1 polycomplexes.

We additionally examined Zip1 polycomplexes in *spo11* strains carrying *P_SCC1_[SMT3]/smt3Δ* (LFT61, Figure S3). Zip1 polycomplexes were displayed by approximately half of the nuclei examined in control (*SMT3+*) or *P_SCC1_[SMT3]/smt3Δ* strains (n>50). Similar to the Zip1 aggregates formed in SUMO-diminished cells with induced Zip1, Zip1 polycomplexes in nuclei from SUMO-diminished *spo11* cells were typically devoid of detectable SUMO.

Taken together, our observations suggest that the elaboration of mature, stable SC structure in budding yeast relies on SUMO. However, since SUMO-free Zip1 might assemble short structures on chromatin and as polycomplex-like aggregates, SUMO may not be required for Zip1 aggregation *per se*. We note, however, that Zip1 structures in SUMO-diminished cells might associate with a level of SUMO that is undetectable under our immuno-staining conditions.

### SUMO-Diminished Cells Exhibit Abnormal Ecm11-MYC Distribution on Meiotic Chromosomes

Ecm11-MYC was recently identified as a SUMOylated protein that both localizes to budding yeast SC in a Zip1-dependent manner and is required for full-length SC formation [29]. Thus, we assessed Ecm11-MYC distribution on meiotic chromosomes in our SUMO-diminished strain backgrounds.

Western blot analysis revealed that overall levels of SUMO-conjugated Ecm11-MYC were dramatically reduced, and overall levels of the unSUMOylated form of Ecm11-MYC were modestly reduced in *P_SCC1_[SMT3]/smt3Δ* strains relative to *SMT3+* strains (both homozygous for an *ndt80* deletion) at multiple time points during a sporulation time course (Figure S4A, B).

Meiotic nuclei from *SMT3+* and *P_SCC1_[SMT3]/smt3Δ ndt80/ndt80* cells that had been sporulating for 24 hours were surface spread and analyzed for Ecm11-MYC, Zip1 and SUMO distribution. In contrast to control cells which exhibited linear stretches of Ecm11-MYC coincident with Zip1 staining, both Zip1 and Ecm11-MYC exhibited a discontinuous, dotty distribution on pachytene stage chromosomes at 24 hours of sporulation in *P_SCC1_[SMT3]/smt3Δ ndt80/ndt80* homozygotes (Figure S4C, D). The Zip1 distribution on chromosomes from SUMO-diminished cells expressing *ECM11-MYC* appeared even more discontinuous, on average, than the Zip1 distribution on chromosomes in SUMO-diminished cells without *ECM11-MYC* (Figure 2), suggesting the possibility that SC assembly in the presence of Ecm11-MYC may be sensitized to SUMO-diminishment. Foci and short stretches of Zip1 and Ecm11 often localized adjacent or overlapping one another, however we often observed Ecm11 staining devoid of Zip1 and Zip1 devoid of Ecm11 in nuclei from *P_SCC1_[SMT3]/smt3Δ* cells.

Next we examined the distribution of both Ecm11-MYC and an unSUMOylatable version of Ecm11, Ecm11(K5R,K101R)-MYC [29], on meiotic chromosomes from SUMO-diminished and control strains in the context of our Zip1 induction system (Figure S5). As described above, inducible *ZIP1* allows us to characterize SC assembly after maximal turnover of SUMO-conjugated proteins (when examining SUMO-diminished genotypes). In these experiments, which are analogous to the experiment shown in Figure 3, *ZIP1* expression under the control of a ß-estradiol-sensitive *P_GAL1_* system is induced after 24–25 hours of sporulation in *ndt80*-arrested cells homozygous for *ECM11-MYC*, and newly induced SCs are analyzed for Zip1 and Ecm11 distribution on chromosomes. After three hours of Zip1 induction in *SMT3+* strains, many full-length SC stretches were observed on meiotic chromosomes and the distribution of Ecm11-MYC was linear and coincident with Zip1 in these SC stretches. In SUMO-diminished strains, on the other hand, both Zip1 and Ecm11-MYC formed foci and very short stretches on meiotic chromosomes. Ecm11-MYC occasionally overlapped Zip1 but often localized independent of a Zip1 entity on these meiotic chromosomes (Figure S5B).

The defects in Ecm11 distribution on meiotic chromosomes that we observe in SUMO-diminished cells, both in an otherwise normal meiosis or in the context of induced Zip1, strengthen the idea that assembly of normal budding yeast SC structure is dependent on SUMO.

Since strains homozygous for the unSUMOylatable *ecm11* allele are defective in Zip1 assembly [29], we expected that Zip1 would fail to assemble SC after *ZIP1* induction in cells homozygous for *ecm11(K5R, K101R)-MYC*. Indeed, after three hours of *ZIP1* induction in *ecm11(K5R, K101R)-MYC* strains, the distribution of both Ecm11(K5R, K101R)-MYC and Zip1 was dotty on chromosomes (Figure S5A). In SUMO-diminished strains carrying inducible *ZIP1* and homozygous for *ecm11(K5R, K101R)*, Ecm11(K5R,K101R)-MYC typically formed foci and short stretches that only occasionally colocalized with the foci and short stretches of Zip1 on meiotic chromosomes (similar to the behavior of Ecm11-MYC in this genetic background).

The colocalization of Ecm11-MYC with Zip1 aggregates that form in SUMO-diminished, Zip1-inducible strains was also assessed. In SUMO-diminished strains carrying the Zip1 inducible system and homozygous for *ECM11-MYC*, 15/21 nuclei exhibited Zip1 aggregates at 3 hours post-induction, and Ecm11-MYC colocalized with 13/15 of the observed Zip1 aggregates (examples are shown in Figure S5B). Zip1 aggregates were reduced in frequency, on the other hand, in SUMO-diminished strains carrying inducible Zip1 and homozygous for an unSUMOylatable *ecm11(K5R, K101R)-MYC* allele (6/24 nuclei exhibited Zip1 aggregates after 3 hours of induction). Four of the six observed polycomplexes displayed no associated Ecm11(K5R, K101R)-MYC protein, whereas in two of the six observed polycomplexes the Ecm11(K5R, K101R)-MYC protein localized peripheral to the Zip1 aggregate (Figure S5B).

### SUMO and Ecm11 Localize to the Central Element of the Budding Yeast SC

The localization of SUMO and Ecm11 at the interface of meiotic pachytene chromosomes depends on Zip1 [19], and loss-of-function analyses ([29], this work) suggest that these components might participate structurally in building SC central region. Immuno-electron microscopy experiments demonstrated that two Zip1 coiled coil units (dimers or tetramers, each predicted to be approximately 50–80 nm in length) span the width of the SC central region, with N termini interacting near one another and with C termini facing the lateral elements/chromosome axes of each homolog [10]. How is SUMO organized relative to Zip1 within the SC?

**Figure 4:**
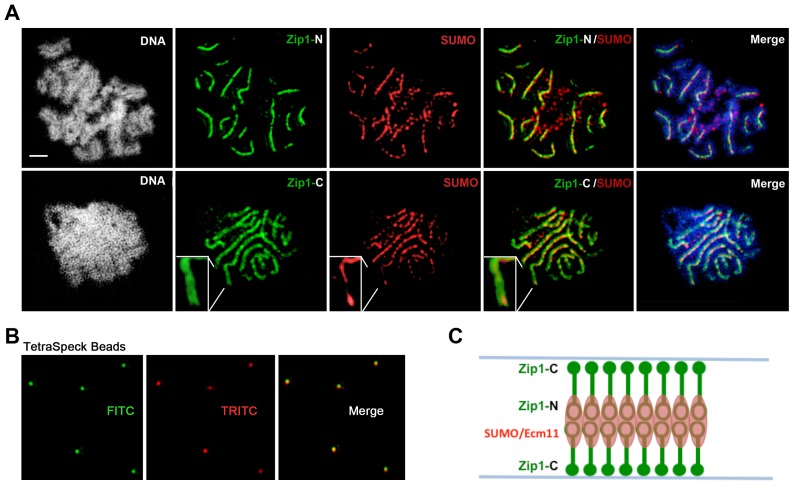
SUMO is positioned at the central element of the budding yeast SC. (A) Surface-spread meiotic nuclei from an *ndt80* homozygous strain (AM447). Top row: nuclear spreads are stained with antibodies that target the Zip1 N terminus (green) and SUMO (red), in addition to DAPI to visualize DNA (white, blue in merged image). Bottom row: nuclei are stained with antibodies targeting a C terminal fragment of Zip1 (green) in addition to SUMO (red) and DAPI (white, blue in merged image). Inset zooms in on a portion of indicated SC. Scale, 1 µm. Images were acquired using a structured illumination microscope (Applied Precision's OMX V4, access kindly provided by Stanford Neuroscience Microscopy Service, Stanford University) that can resolve the SC central element (Zip1-N staining) from the remainder of the SC (where Zip1-C is located). The presence of a slight offset between Zip1-N and SUMO staining patterns can be attributed to a chromatic aberration during imaging, as shown in (B). (B) TetraSpeck Beads (Invitrogen), stained uniformly with both red and green fluorescent dyes, were imaged using the same mount and oil conditions as were used to image meiotic nuclei. The resultant red and green bead patterns failed to completely overlap, indicating that a slight chromatic aberration exists for the imaging conditions used. The cartoon in (C) illustrates a model for budding yeast SC structure, with SUMO and Ecm11 (see Figure 5) positioned at the N termini of Zip1 within the SC central region.

**Figure 5:**
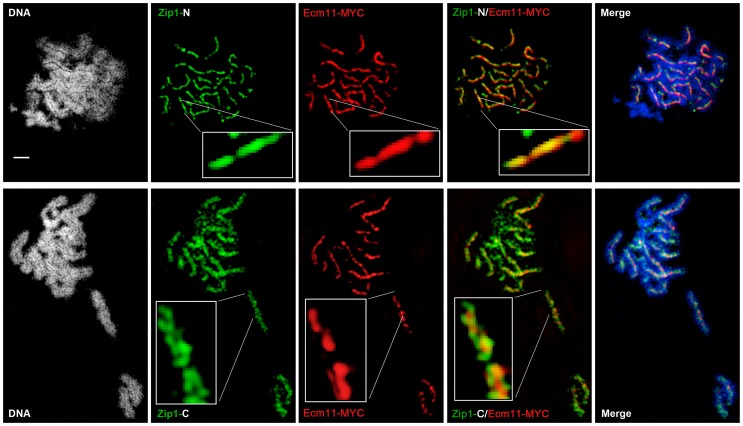
Ecm11-MYC is positioned at the central element of the budding yeast SC. Surface-spread meiotic nuclei from an *ECM11-MYC ndt80* homozygous strain (AM2712). Top row: nuclear spreads are stained with antibodies that target the Zip1 N terminus (green) and MYC (red), in addition to DAPI to visualize DNA (white, blue in merged image). Bottom row: nuclei are stained with antibodies targeting a C terminal fragment of Zip1 (green) in addition to MYC (red) and DAPI (white, blue in merge). Boxed insets show portions of each image with increased zoom. The images were acquired using Applied Precision's V4 OMX structured illumination microscope at Stanford Neuroscience Services (Stanford University) as described in Figure 4. Scale, 1 µm.

We used superresolution microscopy and Zip1 domain-specific antibodies to map the substructural location of SUMO and the Ecm11 protein within the budding yeast SC. Dong and Roeder [10] showed that antibodies targeting an N-terminal fragment of Zip1 label a narrow central domain at the interface of lengthwise-aligned homologs, while antibodies targeting Zip1 C termini decorate a wider domain, reflecting the proximity of Zip1-C to lateral elements. We labeled surface-spread meiotic nuclei with Zip1 domain-specific antibodies in addition to either anti-SUMO (Figure 4), or anti-MYC (on sporulated cells from strains carrying Ecm11-MYC) antibodies (Figure 5). We used Applied Precision's V4 OMX Structured Illumination microscope (access kindly provided by Stanford Neuroscience Services, Stanford University) to visualize labeled proteins on chromosome spreads.

The OMX SIM system produced images with sufficient resolution to recapitulate the results of the earlier immuno-EM study conducted by Dong and Roeder. As shown in Figures 4 and 5, antibodies targeting amino acids 20–139 at Zip1's N terminus [10] decorate a narrow, linear domain at the interface of lengthwise-aligned homologs, while antibodies raised against the 264 C-terminal amino acids of Zip1 [8], [46] exhibit a substantially wider domain of staining, wherein two parallel tracts can often be distinguished (green staining in Figures 4 and 5). Antibodies targeting the meiotic chromosomal axis component, Red1, displayed a pattern of staining that was similar to that of Zip1-C; homolog pairs often displayed parallel Red1 tracks along their lengths (Figure S6). Such positional information is completely absent from images of immuno-stained SC taken using conventional fluorescence microscopy followed by deconvolution on Applied Precision's Deltavision RT microscope system (Figure S7).

Moreover, SUMO or the SUMOylated SC component, Ecm11 [29], localize to a narrow domain that coincides with the position of Zip1's N termini, between the parallel tracks decorated by anti-Zip1-C (Figure 4 and 5). We note that a slight shift in the location of SUMO or Ecm11-MYC relative to the Zip1 N terminal domain can be accounted for by a chromatic aberration; the existence of such an aberration is demonstrated by the slight offset of red and green images produced by TetraSpeck beads, which are uniformly labeled with both green and red fluorophores (Figure 4B).

As depicted in the cartoon in Figure 4C, our structured illumination data independently confirm an organization of Zip1 within SC that was deduced using Zip1 domain-specific antibodies in conjunction with electron microscopy and furthermore demonstrate that the smaller Ecm11 and SUMO proteins assemble near Zip1's N termini. These findings thus reveal the first known components of the budding yeast SC “central element” domain.

### Budding Yeast Central Element Proteins Exhibit Ongoing Incorporation into Full-Length SC

We next asked whether SUMO and Ecm11 share the dynamic behavior that Zip1 exhibits within full-length SC. Using inducible *ZIP1-GFP*, we have demonstrated that Zip1 continually incorporates into previously deposited, full length SC during meiotic prophase [46]. We observed that post-synapsis Zip1-GFP initially decorates full-length SC as foci and, over time, develops into a linear pattern that coincides with previously deposited SC. We used an analogous induction strategy to ask whether SUMO and Ecm11 exhibit post-synapsis incorporation into full length SC.

**Figure 6:**
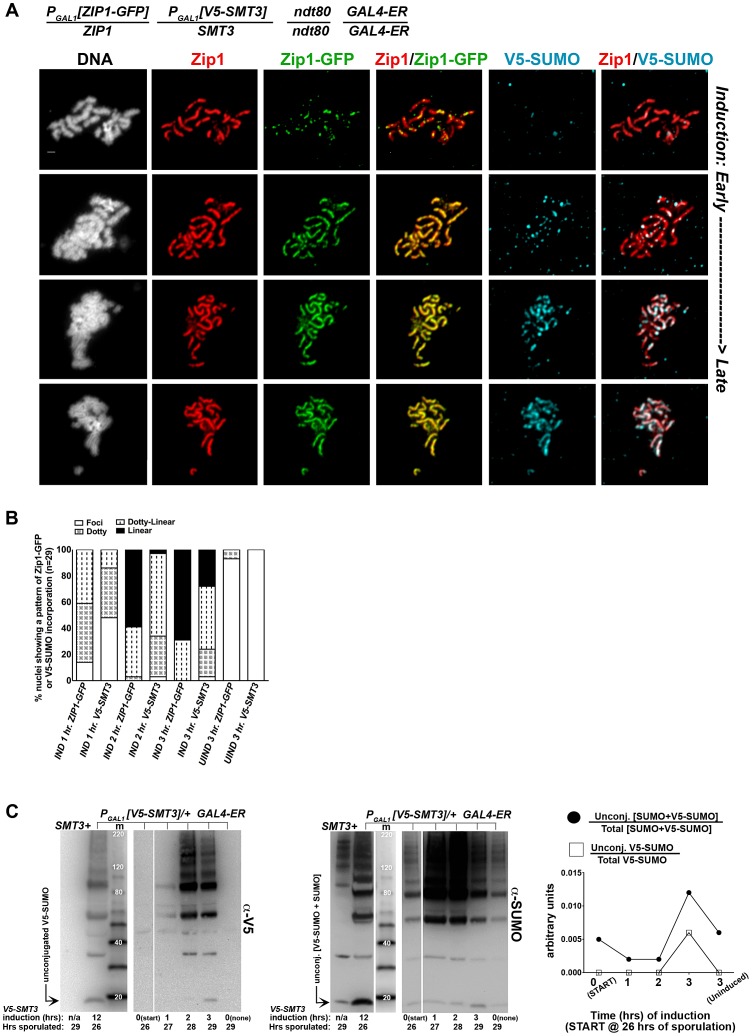
V5-SUMO continuously incorporates into previously established, full-length SC during meiotic prophase. The strain in (A), AM2905, is homozygous for an *ndt80* mutation, carries two copies of the *GAL4-ER* transgene, and is heterozygous for both *ZIP1-GFP* and *V5-SMT3* under the transcriptional control of the *P_GAL1_* promoter. Most *ndt80* mutant meiotic nuclei in this strain background exhibit full-length SC by 24 hours of sporulation. Each strain was induced to express *ZIP1-GFP* and *V5-SMT3* at 26 hours of sporulation, and then assessed at 1, 2 and 3 hours following induction. Uninduced cells were also assessed at 3 hours post-induction. Representative images of surface-spread nuclei from induced cells are shown, with top rows showing nuclei with lower levels of induced SC component incorporation and bottom rows showing SC component incorporation that is almost completely coincident with Zip1. Staining is as follows: DAPI (DNA), white; Zip1 (and/or Zip1-GFP), red; Zip1-GFP, green, V5-SUMO, blue. Scale, 1 µm. The stacked column graph in (B) indicates the fraction of nuclei with full-length Zip1 that exhibited None/Foci (open), Dotty (boxed), Dotty-linear (dotted lines), or Linear (solid) patterns of induced Zip1-GFP or V5-SUMO patterns on previously-established SC (n = 30). In (C), membranes with immobilized proteins from lysates of samples taken at the induction (START, 26 hours of sporulation), 1 hour, 2 hour and 3 hour post-induction, and a 3 hour uninduced sample were stained with anti-V5, then stripped and re-probed with anti-SUMO. Lane one contains sample from a strain containing no V5 tag, lane 2 contains AM2905 that has been induced for 12 hours, lane 3 contains MagicMarker protein standards (kDa) (Invitrogen), and lanes 4–8 contain AM2905 lysates at various time points. Unconjugated V5-SUMO and SUMO are indicated in the image. Graph at right plots the relative level of unconjugated V5-SUMO to total V5-SUMO (open boxes) and of unconjugated [V5-SUMO+SUMO] to total [V5-SUMO+SUMO] (closed circles) in each of lanes 4–8.

We first created an *ndt80* homozygous strain carrying one copy of inducible V5-tagged *SMT3*, (*P_GAL1_[V5-SMT3]*), and one copy of inducible *ZIP1-GFP* (*P_GAL1_[ZIP1-GFP]*) in addition to one endogenous *ZIP1* and *SMT3* allele. This strain (AM2905) also contains the *GAL4-ER* gene, which induces expression from *P_GAL_* promoters upon exposure to ß-estradiol. After 26 hours in sporulation media, most AM2905 cells have deposited full-length SC, but are blocked from progressing further through meiosis due to the absence of the Ndt80 transcription factor. We induced *ZIP1-GFP* and *V5-SMT3* expression in AM2905 cells that had been sporulated for 26 hours, and then prepared chromosome spreads from induced cultures after one, two or three hours. We also surface-spread cultures that were uninduced after three hours. We assessed the localization of Zip1, Zip1-GFP and V5-SUMO using antibodies against each protein or protein tag. As previously described [46], the majority of full-length SCs previously built with untagged Zip1 and untagged SUMO (not shown) exhibit Zip1-GFP foci and linear assemblies after one hour of induction (Figure 6A, B). After three hours of induction, most chromosome spreads exhibit linear Zip1-GFP that partially or completely coincides with the previously deposited SC. On the other hand, few nuclei exhibited robust V5-SUMO staining after one hour of induction, although approximately half of the nuclei exhibited a dotty pattern of V5-SUMO on chromosomes (Figure 6B). After two hours of induction, many chromosome spreads exhibited foci and some short linear stretches of V5-SUMO, and at three hours of induction approximately 30 percent of nuclei exhibited V5-SUMO linear stretches coincident with nearly the full length of SC. These observations demonstrate that V5-SUMO incorporates into previously deposited SC in a similar manner as Zip1-GFP, but perhaps with a different rate of incorporation.

The lag in V5-SUMO post-synapsis incorporation (relative to Zip1-GFP post-synapsis incorporation) does not appear to be due to a delay in the formation of V5-SUMO conjugates within meiotic cells *per se*. Induced, unconjugated (“free”) V5-SUMO comprised only a small fraction of total V5-SUMO signal in lysates from cells even at one hour post-induction; V5-SUMO remained a small fraction of total V5-SUMO at two and three hours of induction, and free untagged SUMO was detected as a similarly small fraction of total SUMO signal in the same samples (Figure 6C). We discuss potential explanations for this lag in V5-SUMO appearance on SCs in the Discussion (see below).

**Figure 7:**
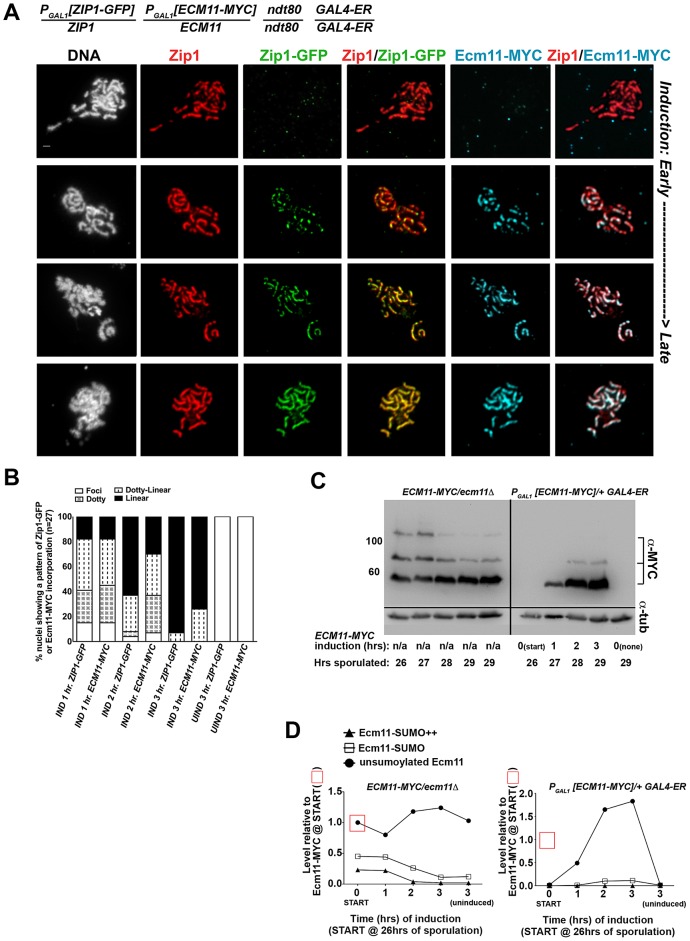
Ecm11-MYC continuously incorporates into previously established, full-length SC during meiotic prophase. The strain in (A), AM2865, is homozygous for an *ndt80* mutation, carries two copies of the *GAL4-ER* transgene, and is heterozygous for both *ZIP1-GFP* and *ECM11-MYC* under the transcriptional control of the *P_GAL1_* promoter. Each strain was induced and assessed as described in Figure 6. Representative images of surface-spread nuclei from uninduced or induced cells are shown; staining is as follows: DAPI (DNA), white; Zip1 (and/or Zip1-GFP), red; Zip1-GFP, green, Ecm11-MYC, blue. Scale, 1 µm. The stacked column graph in (B) indicates the fraction of nuclei with full-length Zip1 that exhibited None/Foci (open), Dotty (boxed), Dotty-linear (dotted lines), or Linear (solid) patterns of induced Zip1-GFP or Ecm11-MYC patterns on previously-deposited SC (n = 30). In (C), membranes with immobilized proteins from lysates of *ECM11-MYC/ecm11Δ ndt80/ndt80* (AM2892) samples (left) and AM2865 (right) taken at the induction START (26 hours of sporulation), 1 hour, 2 hour and 3 hour post-induction, and a 3 hour uninduced sample were stained with anti-MYC, stripped and re-probed with anti-α-tubulin. Hemizygous *ECM11-MYC* lysates were included as a reference for baseline Ecm11 levels at each timepoint. Numbers at left give molecular weight positions (kDa). Graphs in (D) at right plot the relative levels of unSUMOylated Ecm11-MYC (closed circles), SUMOylated Ecm11-MYC (open squares) and multi-SUMOylated Ecm11-MYC (closed triangles) in each lane (at each timepoint of the time course experiments); the level of unSUMOylated Ecm11-MYC at 26 hours in Ecm11-MYC/*ecm11Δ* is set at 1. Relative levels between lanes were normalized using the tubulin staining.

We next analyzed the post-synapsis dynamics of Ecm11-MYC using a strain (AM2865) analogous to AM2905 but carrying one copy of inducible *ECM11-MYC* instead of inducible *V5-SMT3*. We performed the induction as described above and stained for the presence of Zip1, Ecm11-MYC, or GFP using antibodies against each protein or protein tag. Analysis of induced Ecm11-MYC protein (Figure 7C, D) indicated that induced Ecm11-MYC rose to approximately 1.5 times the level of baseline Ecm11 (the level of Ecm11 present in hemizygous *ECM11/ecm11Δ*strains) by two hours post-induction, and reached nearly twice the level of baseline Ecm11 by three hours post-induction. Figure 7A shows examples of uninduced (top row) and induced nuclear spreads. We observed that, similar to post-synapsis Zip1-GFP and V5-SUMO, induced Ecm11-MYC incorporates into previously deposited SCs as both foci and, later, as linear assemblies that eventually coincide with the full length of the SC. Unlike V5-SUMO, initial post-synapsis incorporation of Ecm11-MYC typically appeared to the same extent as post-synapsis incorporation of Zip1-GFP in individual nuclei at each time point examined (Figure 7A, B).

These findings demonstrate that central element proteins (such as SUMO and Ecm11) and the transverse filament protein, Zip1, exhibit a similar post-synapsis dynamic of ongoing incorporation into full-length SC, consistent with a model in which Zip1, Ecm11 and SUMO (and/or SUMOylated Ecm11) represent core building blocks of the SC structure.

### UnSUMOylatable Ecm11 Is Capable of Incorporating into SC Structure

**Figure 8:**
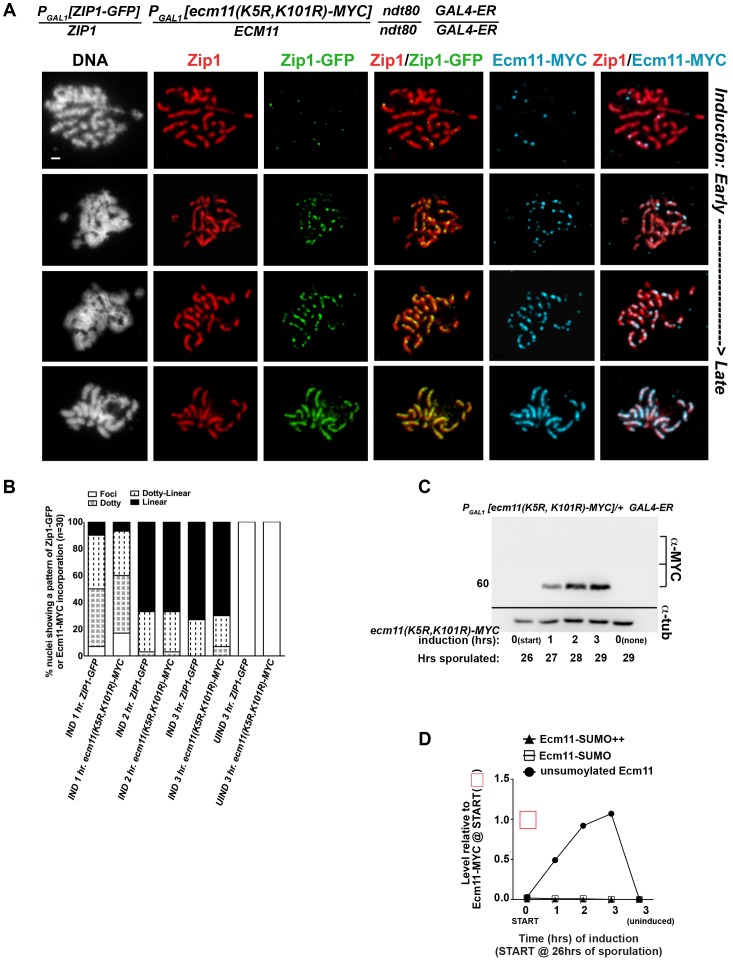
Ecm11(K5R,K101R)-MYC continuously incorporates into previously established, full-length SC during meiotic prophase. The strain in (A), AM2910, is homozygous for an *ndt80* mutation, carries two copies of the *GAL4-ER* transgene, and is heterozygous for both *ZIP1-GFP* and *ECM11(K5R,K101R)-MYC* under the transcriptional control of the *P_GAL1_* promoter. Each strain was induced and assessed as described in Figure 6. Representative images of surface-spread nuclei from uninduced or induced cells are shown; staining is as follows: DAPI (DNA), white; Zip1 (and/or Zip1-GFP), red; Zip1-GFP, green, Ecm11(K5R,K101R)-MYC, blue. Scale, 1 µm. The stacked column graph in (B) indicates the fraction of nuclei with full-length Zip1 that exhibited None/Foci (open), Dotty (boxed), Dotty-linear (dotted lines), or Linear (solid) patterns of induced Zip1-GFP or Ecm11(K5R,K101R)-MYC patterns on previously-established SC (n = 30). In (C), membranes with immobilized proteins from lysates of AM2910 cells taken at the induction (START, 26 hours of sporulation), 1 hour, 2 hour and 3 hour post-induction, and a 3 hour uninduced sample were stained with anti-MYC, stripped and re-probed with anti-α-tubulin. Numbers at left give molecular weight positions. Graph in (D) plots the level of unSUMOylated Ecm11(K5R,K101R)-MYC (closed circles), SUMOylated Ecm11(K5R,K101R)-MYC (open squares) and multi-SUMOylated Ecm11(K5R,K101R)-MYC (closed triangles) at each timepoint during the induction, relative to the level of unSUMOylated Ecm11-MYC at 26 hours in Ecm11-MYC/*ecm11Δ* samples (from western in Figure 7D). Ecm11 levels were made comparable with blot in Figure 7C by setting the level of Ecm11(K5R,K101R) signal in lane 2 (27 hour timepoint, 1 hour of induction) to the level of Ecm11-MYC signal timepoint in lane 7 (27 hour timepoint, 1 hour of induction) of blot in Figure 7C. Relative levels between lanes were normalized using the tubulin staining.

We also assessed the post-synapsis dynamics of an unSUMOylatable version of Ecm11, Ecm11(K5R, K101R)-MYC. We performed the induction and staining as described above, using a strain (AM2910) carrying one copy of inducible *ecm11(K5R, K101R)-MYC* (and one copy of inducible *ZIP1-GFP*). Analysis of induced Ecm11(K5R, K101R)-MYC protein levels (Figure 7C, D and 8C, D) indicated that induced unSUMOylated Ecm11-MYC rose to approximately that of baseline Ecm11 between two and three hours post-induction.

Figure 8A shows examples of uninduced (top row) and induced nuclear spreads. Somewhat surprisingly, induced Ecm11(K5R, K101R)-MYC incorporated into previously deposited, full length SCs as both foci and, later, as linear assemblies that eventually coincide with the full length of the SC (Figure 8A, B). The timing of incorporation of induced Ecm11(K5R, K101R)-MYC into previously deposited SC was not obviously different from that of induced wild-type Ecm11-MYC.

The observation that unSUMOylated Ecm11 incorporates into previously established SC was unanticipated since homozygous *ecm11(K5R, K101R)* mutants fail to build robust Zip1 assemblies on chromosomes [29]. Consistent with the idea that unSUMOylatable Ecm11 can incorporate into SCs built with pre-existing Ecm11, we also observed that Ecm11(K5R, K101R)-MYC decorates the lengths of SCs in strains heterozygous for *ecm11(K5R, K101R)-MYC* over a wild type (untagged) *ECM11* allele (Figure S8). The spore viability of *ecm11(K5R, K101R)-MYC/ECM11+* heterozygotes (92%, n = 304 spores) was indistinguishable from that of *ECM11-MYC/ECM11+* heterozygotes (93%, n = 244 spores), suggesting that the incorporation of this unSUMOylatable Ecm11 protein does not have a dominant negative effect on meiotic events. It is noteworthy, in light of these findings, that the majority of Ecm11-MYC detected in otherwise wild-type meiotic cell extracts is apparently unSUMOylated (based on size).

We next investigated the pattern of incorporation of unSUMOylatable Ecm11-MYC protein within incipient SC stretches. We analyzed the localization of Ecm11(K5R, K101R)-MYC on the short Zip1 stretches observed after one hour of induction in Zip1-induction strains carrying one copy of *ECM11(K5R, K101R)-MYC* in *trans* with a wild-type, untagged *ECM11* allele (Figure S9). The existence of subdomain(s) of Ecm11(K5R, K101R)-MYC within an incipient SC stretch would suggest that unSUMOylated Ecm11-MYC incorporates only after a significant stretch of SUMOylated Ecm11. However, we were unable to detect distinct subdomains of Ecm11(K5R, K101R)-MYC within short Zip1 stretches in these analyses. Instead, Ecm11(K5R, K101R)-MYC colocalized with induced Zip1 assemblies of all sizes with uniform staining (Figure S9).

Thus, although SUMOylatable Ecm11 is required for the formation of SC, Ecm11-MYC need not be SUMOylated for its incorporation into SC structures.

### The Relative Abundance of SUMO and Ecm11-MYC Proteins Correlates with Zip1 Abundance in SC

We previously demonstrated that budding yeast SCs accumulate a progressively larger abundance of Zip1 during meiotic prophase progression, and that Zip1 abundance within SC correlates with *ZIP1* copy number [46]. In order to ask whether SUMO and Ecm11-MYC proteins are also at increased levels within SCs containing a larger abundance of transverse filament protein, we measured Zip1-YFP, SUMO and Ecm11-MYC protein levels in SCs from strains with one, two, and four copies of *ZIP1-YFP*. We assessed the relative abundance of each protein by first defining a small domain of well-spread SC, and then determining the optimal exposure time for acquiring an image within linear range (shorter exposure times reflect a larger abundance of protein, and *vice versa*) (Figure S10A). When images containing small sections of SC are plotted such that their Zip1-YFP exposure time is reflected on the y axis and their SUMO exposure time is displayed on the x axis, an overall trend is observed in which relatively shorter exposure times for Zip1-YFP (i.e. SCs with a larger abundance of Zip1-YFP) correlate with relatively shorter exposure times for SUMO. (Exposure time data from individual images taken of SC from one, two and four copy *ZIP1-YFP* strains, plotted together on the same graph, are shown in Figure S10B.) These data indicate that SUMO abundance is larger in SCs with greater Zip1 abundance, relative to the SUMO abundance in SCs with less Zip1.

When Ecm11-MYC exposure times were plotted against Zip1-YFP exposure times (Figure S10C) a similar positive correlation was found, especially within shorter Zip1-YFP exposure times (greater Zip1-YFP abundance). The correlation coefficients for both Zip1-YFP with SUMO exposure times and Zip1-YFP with Ecm11-MYC exposure times are statistically significant (P≤0.0004, Figure S10B legend). Relative to the SUMO and Zip1-YFP correlation, however, a wider range of Ecm11-MYC exposure times (Ecm11-MYC abundance) was apparent in SC segments exhibiting Zip1-YFP exposure times above 0.5 seconds (Figure S10C). One explanation for the lower degree of correlation between Ecm11 and Zip1-YFP levels in SCs with lower Zip1-YFP abundance could be that Ecm11 both incorporates and *exits* from the full-length SC. Under this model the abundance of Ecm11-MYC would be expected to grow more slowly than the abundance of Zip1-YFP or SUMO within SC. An alternative explanation could be that proportionally fewer Ecm11-MYC molecules incorporate into a given unit of SC as compared to SUMO and Zip1-YFP. This latter idea is consistent with the fact that each transverse filament unit that spans the width of the SC is comprised of two (or a multiple of two) dimers of Zip1-YFP, which themselves may be linked by perhaps just one or two Ecm11 monomers. Furthermore, the Ecm11 protein has the capacity to be conjugated to more than one SUMO molecule [29]. Thus it is conceivable that proportionally fewer molecules of Ecm11 versus SUMO and Zip1 comprise a single “unit” of SC structure.

## Discussion

### SUMO Likely Functions as a Structural Component of the Budding Yeast SC

A role for SUMO in budding yeast SC biology has long been suspected based on the fact that SUMO exhibits Zip1-dependent localization to meiotic chromosomes and based on the SC assembly defects exhibited by mutants in which a particular SC-localized protein (such as Ubc9 or Ecm11) cannot be SUMOylated [19], [29], [47]. Here we directly reveal a role for SUMO in the assembly of mature SC in budding yeast, by analyzing meiotic mutants with reduced SUMO levels. We show that in the context of an inducible-SC system (using strains homozygous for inducible *ZIP1*), SUMO diminishment results in a dramatic SC assembly defect that resembles *ecm11(K5R, K101R)* (and other synapsis-defective) mutants [29]. This SC assembly defect, observed under the most robust SUMO-diminished conditions, leaves open the possibility that SUMO is involved exclusively in initial stages of SC elaboration. Notably, in meiotic nuclei that have normal *ZIP1* regulation and overall milder SUMO reduction we also observed a discontinuous pattern of SC assembly on most pachytene stage chromosomes. This latter, highly penetrant but less dramatic SC phenotype is consistent with the idea that SC requires SUMO on an ongoing basis for its proper assembly and/or maintenance.

Furthermore, we demonstrate that SUMO and Ecm11 exhibit ongoing incorporation into previously deposited SC and that these components grow in abundance (SUMO to a greater extent than Ecm11) as SC accumulates additional Zip1 content. Together with the fact that SUMO exhibits a linear staining pattern coincident with transverse filaments along the length of SCs, these findings suggest that SUMO is a core structural component of the budding yeast SC.

If SUMO-diminishment causes aberrant SC structures, why is the spore viability of SUMO-diminished strains only slightly decreased relative to control strains? There are at least three explanations that could account for the high viability of spores produced by SUMO-diminished strains. First, it is important to note that SUMO-diminished cells do exhibit SC structures (albeit discontinuous) in cells carrying endogenous (as opposed to inducible) *ZIP1*; such aberrant SC structures may be capable of executing the chromosome segregation functions required for high spore viability. Second, we observed that interhomolog crossover recombination is modestly but significantly elevated in SUMO-diminished cells; it is possible that a set of “extra” crossovers protect SUMO-diminished cells from the chromosome segregation errors that might arise as a consequence of aberrant SC. Finally, it should be noted that *ecm11* mutants, which show defective SC assembly, nevertheless exhibit significantly higher spore viability than other synapsis-defective mutants [29]. We wonder therefore if synapsis proteins such as Zip1, Zip2 and Zip4 function in Ecm11/SUMO-independent chromosome segregation activities outside of their role in SC structure. An obvious candidate activity with regard to this idea is the Zip1-dependent “centromere tethering” phenomenon, where centromeres associate in two-by-two pairs after SC disassembly [48], [49].

### SUMO and Ecm11: The First Identified Components of the SC Central Element in Budding Yeast

While SUMO's position within the larger architecture of the SC was not known until now, based on the observations that 1) a small domain at Zip1's C-terminus has the capacity to interact with SUMO in a yeast two hybrid system [27], [50] and 2) a subset of the Red1 chromosome axis protein is SUMOylated during meiotic prophase [27], one widely-cited model proposed that SUMO associates with Zip1's C terminal domain along the length of fully assembled SC [25], [26], [28], [50].

Using superresolution microscopy in conjunction with Zip1 domain-specific antibodies, we demonstrate that instead of the scenario mentioned above, SUMO and Ecm11 lie in close proximity to Zip1-N termini within the mature SC structure. These findings make SUMO and Ecm11 the first known components of the SC central element, a subdomain at the midline of SC central region that is sometimes apparent as an electron dense entity in ultrastructural images of synapsed chromosomes.

As the defect in SC assembly exhibited by our SUMO-diminished meiotic cells closely resembles that observed for strains expressing an unSUMOylatable *ecm11* allele [29], perhaps SUMOylated Ecm11 is the predominant source of SUMO within SC central element. However it remains possible that additional SC central element proteins that act in parallel or downstream of Ecm11 to promote SC assembly are also SUMOylated (e.g. see [47]).

SUMO has not yet been identified as a core component of SC structure outside of budding yeast [28], [51]. However, SUMO and Ecm11 join a growing list of central element proteins from different organisms that are critical for SC assembly. Mutants that are missing central element proteins in both *Drosophila* and mouse exhibit defects in establishing stable SC [15], [16], [18], [22], [23], [24]. Thus, perhaps a conserved feature of SC assembly is the role that the central element plays in properly organizing the coiled-coil proteins that form the transverse filaments.

### The Spatial Relationship between SUMO and Zip1 on Meiotic Chromosomes

**Figure 9:**
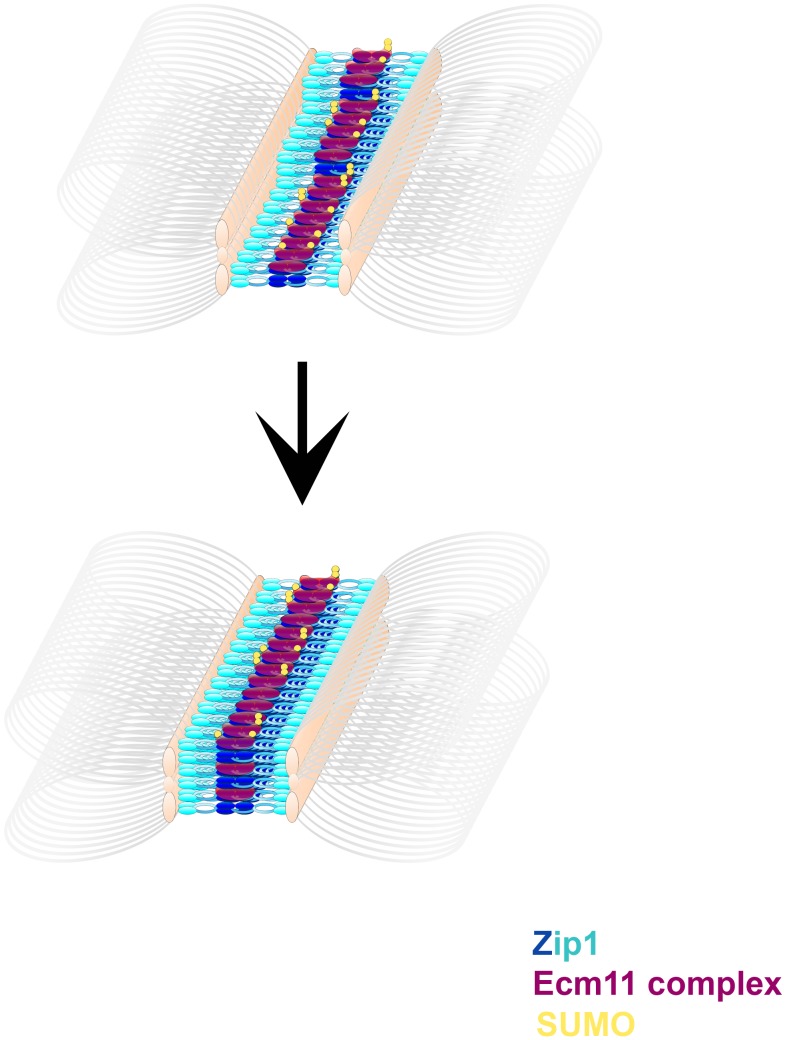
A model describing the multimeric assembly of Zip1, Ecm11 and SUMO within SC. Cartoon images shows possible intermediate steps in a dynamic SC assembly process. Zip1 (blue, darker blue at Zip1-N termini) units are stacked with Ecm11 complex proteins (purple) arranged near Zip1 N termini. Ecm11 components are SUMOylated (yellow) to varying extents. Multiple layers of this basic arrangement of Zip1, Ecm11 and SUMO comprise more mature SC structures.

A prior study found that an N terminal ∼160 amino acid fragment of Zip1 (representing nearly the entire N terminal globular domain) is dispensable for normal SC [52], seemingly at odds with the idea that the budding yeast SC central element plays a key role in SC assembly. However, this study also characterized three deletions within the N terminal region of Zip1's coiled-coil; strains carrying each of these *zip1* deletion alleles exhibited defective SC assembly. Perhaps it is this N terminal portion of the Zip1 coiled-coil that interacts, directly or indirectly, with central element components in order to organize the mature SC structure. One way to think about such an interaction is that central element proteins, such as a complex containing SUMO and/or SUMOylated Ecm11, stabilize a self interaction between two N terminal portions of Zip1 coiled-coil units that lie head-to-head within SC central region ([52], Figure 9). Alternatively, antiparallel Zip1 units that span the SC may each independently interact with a “bridge” of central element proteins. Either model would account for the fact that deletions within Zip1's coiled-coil region narrow the SC diameter but similarly-sized deletions in Zip1's N terminal region, prior to the coiled-coil domain, assemble SC with a normal width [52].

Our observation that the predominant SUMO signal within budding yeast SC lies closer to Zip1 N termini does not rule out the possibility that a subset of Zip1 protein also interacts with SUMO or SUMO polymeric chains through its C terminal SUMO Interaction Motif (SIM), as has been proposed [25], [26], [27], [28]. Such an interaction might mediate a chromosomal process transiently and/or at discrete sites, such as synapsis initiation or interhomolog recombination (as has been suggested based on the phenotype of a strain carrying an unSUMOylatable *red1* allele [27]), and thus escape detection in cytological preparations. Indeed, the fact that only a small fraction of total Red1 protein is SUMOylated during meiosis [27] is consistent with the idea that SUMOylated Red1 mediates functions at a limited number of discrete sites on meiotic chromosomes (instead of along the full SC length). Perhaps the elevated interhomolog crossover levels that we observed in SUMO-diminished strains are due to a reduced level of SUMOylated Red1. Alternatively the observed increase in interhomolog crossover recombination in SUMO-diminished strains might be a consequence of defective SC structures or the misregulation of a SUMOylated component of the recombination machinery.

### The Stoichiometry and Timing of SC Component Assembly

Our analysis of SUMO-diminished strains is consistent with a model in which SUMO promotes and/or maintains the higher-order assembly and stability of mature SC structure. We have observed that SUMO abundance increases within SCs in proportion to Zip1 abundance. In contrast, Ecm11's abundance did not grow to the same extent as SUMO within SCs containing larger amounts of Zip1. These data raise the possibility that multiple SUMO (and multiple Zip1) proteins accompany a single Ecm11 protein in an SC unit.

Some of our observations also suggest that SUMO might not incorporate into SC structures with the same kinetics as Ecm11. First, we observed a distinct lag in the appearance of induced V5-SUMO, relative to the appearance of induced Zip1-GFP and induced Ecm11-MYC into previously established SCs. One explanation for this “lag” in post-synapsis V5-SUMO incorporation could be that induced V5-SUMO does not become efficiently conjugated to proteins, perhaps because it is competing with a larger pool of untagged SUMO. However, our analysis of free V5-SUMO versus V5-SUMO conjugates in these strains suggests that V5-SUMO molecules become conjugated to meiotic proteins efficiently after their translation (Figure 6C).

The lag in detectable post-synapsis V5-SUMO relative to Zip1-GFP and Ecm11-MYC on SCs could be the result of a technical limitation, such as a difference in the efficiency of V5-SUMO translation relative to Ecm11-MYC, or a difference in binding affinity of anti-V5 versus anti-GFP on meiotic chromosome spreads. However, if this lag truly reflects a slower incorporation rate for SUMO versus other SC components, it suggests that either 1) SUMOylation of SC components can be an event that occurs subsequent to the incorporation of the unSUMOylated versions of SUMO targets, or 2) that only a subset of Ecm11 or other SUMOylated SC proteins are SUMOylated when they incorporate into SC.

Our analysis of strains carrying a tagged *ecm11(K5R, K101R)* allele shows that, in fact, unSUMOylated Ecm11 readily incorporates into nascent as well as previously-deposited SC. SUMOylated Ecm11, however, is likely required for generating stable SC as strains homozygous for an *ecm11(K5R, K101R)* mutation fail to exhibit extensive SC [29]. These data are consistent with the idea that unSUMOylated Ecm11 may assemble into SC, but that SUMOylated Ecm11 promotes the reinforcement or stabilization of SC proteins near the N termini of Zip1.

Figure 9 displays a cartoon model for SC establishment based on the observations provided in this study. We propose that transverse filaments (Zip1 dimers) are organized near their N termini by a complex of proteins including Ecm11 and SUMOylated Ecm11, but that SUMOylated, multi-SUMOylated, and unSUMOylated versions of Ecm11 exist in the SC structure at any given time. We are intrigued with the idea that SUMOylation of Ecm11 may occur subsequent to Ecm11's positioning within an intermediate, immature SC structure, however it is also possible that Ecm11's SUMOylation status is established prior to, and cannot be modified subsequent to, its incorporation into SC. Regardless of the timing of SUMOylation, we propose that the stoichiometry of SC maturation is such that several SUMO and Zip1 proteins (eventually) accompany a smaller number of Ecm11 molecules within each SC unit. Thus as the SC builds layers, SUMO and Zip1 abundance increase to a greater extent than Ecm11 protein within the budding yeast SC.

## Methods

### Genetics

Strains used in this study are isogenic with BR1919-8B [53], and their genotypes are listed in Table S1. Yeast genetic manipulations were carried out via standard procedures. Two consecutive transformations on diploid cells created the SUMO-diminished strains used in this study. First, we replaced the *SMT3* ORF with a dominant drug resistance cassette (*hphMX4* or *natMX3* [54]). Next a *kanMX4-P_SCC1_* promoter cassette was inserted upstream of the remaining *SMT3* gene. Strains were verified by assessing SUMO levels on a western blot or by immuno-staining.

Genetic map distances of three intervals on chromosome III in SUMO-diminished and control backgrounds were calculated according to the following: 100(*r/t*), where *r* = the number of colonies carrying a chromosome which is recombinant in the interval and *t* = the total number of colonies assessed. Only tetrads with 2 viable spores were used to determine genetic recombination data. Standard error (S.E.) values for random spore analysis were calculated according to the formula: 100(√(r/t)(1-r/t)/t) [55]. Map distances calculated using standard tetrad analysis, as per Perkins [56], were generated using the Stahl lab online tools: http://molbio.uoregon.edu/~fstahl/.

### Cytological Analysis and Imaging

To determine the kinetics of multinucleate formation in Figure 1A, cells were fixed in 50% ethanol and frozen at −20°C prior to staining with DAPI [57].

Meiotic chromosome spreads, staining and imaging were carried out as previously described [46]. The following primary antibodies were used: chicken anti-GFP (1∶100) (Abcam), mouse anti c-MYC (1∶200) (9E10.3 Abcam and Invitrogen), affinity purified rabbit anti-Zip1 (1∶100) (raised at YenZym Antibodies, LLC, against a C terminal fragment of Zip1 as described in [8]), affinity purified guinea pig anti-SUMO (1∶200), rabbit anti-Red1 (1∶100) (kind gifts of G.S. Roeder, [19]), rabbit anti-V5 (1∶100) (Abcam) and rat anti-alpha tubulin (1∶200) (Santa Cruz). Secondary antibodies were obtained from Jackson ImmunoResearch and used at a 1∶200 dilution. All secondary antibodies for Structured Illumination microscopy were conjugated to Alexa Fluor fluorescent dyes (Alexa Fluor 488, AlexaFluor 568, AlexaFluor 594, Molecular Probes Inc.).

Microscopy and image processing was carried out using a Deltavision RT imaging system (Applied Precision) adapted to an Olympus (IX71) microscope. The structured illumination microscopy presented in Figures 2 and 3 were carried out using Applied Precision's V4 OMX Structured Illumination Microscope system at Stanford University's Neuroscience Services facility, while the imaging done for Figure S2 was carried out on Applied Precision's OMX Blaze Structured Illumination Microscope system at The Rockefeller University's Bio-Imaging Resource Center.

### Induction Experiments

The *TRP1::P_GAL1_* promoter cassette was placed upstream of the *ZIP1*, *V5-SUMO*, or *ECM11-MYC* ORFs by directed transformation of a PCR product. pKB80 (*GAL4.ER::URA3*) was integrated at *ura3* to introduce the chimeric protein that responds to β-estradiol and activates *P_GAL1_* promoters [58]. Strains were handled and induced as described in [46].

### Western Blot

Protein pellets were isolated from 5 mL of sporulating cell culture by TCA precipitation as in [19]. The final protein pellet was suspended in 2× Laemmli sample buffer supplemented with 30 mM DTT, at a concentration of ∼10 µg/µl. Protein samples were heated for 10 minutes at 65°, centrifuged at top speed and ∼150 µg was loaded onto either an 8% (for detecting Zip1or Ecm11-MYC) or a 4–20% gradient (for detecting SUMO and V5-SUMO) polyacrylamide/SDS gel. PDVF membranes were prepared according to manufacturer's (Bio-Rad) recommendation, equilibrating with Towbin buffer for 15 minutes after methanol wetting. Transfer of proteins to PDVF membranes was done following Bio-Rad Protein Blotting Guide for tank blotting using Towbin Buffer; stir bar and ice pack were used at 60 V for transfer. SUMO and V5-SUMO blots were transferred for 40 minutes whereas the Ecm11-myc and Zip1 blots were transferred for 1 hour. Ponceau S was used to detect total protein on the PVDF membrane after transfer. Guinea pig anti-SUMO (kind gift of Shirleen Roeder's lab) and rabbit anti-V5 (Abcam) were used at 1∶500 dilution. Rat anti-tubulin (Santa Cruz), mouse anti-MYC (9E10.3, Invitrogen), and rabbit anti-Zip1 were used at 1∶5000–10000, 1∶2500 and 1∶5000 dilutions respectively. Incubations in primary antibody were performed overnight at 4°C. HRP-conjugated AffiniPure Donkey anti-rabbit and anti- guinea pig, as well as goat anti-mouse (JacksonImmunoResearch) and goat anti-rat (Santa Cruz) were used at 1∶5000 in TBS-T for 1 hour at RT. Amersham ECL Prime Western Blotting Detection Reagent was used to visualize antibodies on the membranes; a Syngene G:Box was used to detect chemiluminescence and the Syngene GeneTools program was used to analyze the data. Membranes were stripped according to the ECL Prime kit protocol.

### Statistical Analyses

All statistical analyses were performed using GraphPad InStat software.

## Supporting Information

Figure S1 SUMO-diminished strains exhibit delayed meiotic spindle formation. (A) Anti-tubulin and anti-Red1 (not shown) sera were applied to surface-spread nuclei from sporulating cultures of control (LY35) and SUMO-diminished (*P_SCC1_[SMT3]/smt3Δ*, LFT62) cells. Examples of the morphology of tubulin structures (red) in meiotic nuclei (DNA, white) at pachytene, diplotene, MI and MII stages from wild type and *P_SCC1_[SMT3]/smt3Δ* cells are shown. Note, the microtubules that bridge the duplicated spindle pole bodies in *P_SCC1_[SMT3]/smt3Δ* nuclei during post-pachytene stages were frequently diminished. Scale, 1 µm. (B) shows the fraction of surface-spread nuclei that were at early meiotic prophase (leptotene - midpachytene), late pachytene-diplotene, or MI/MII for each time point, assessed based on DAPI, Red1 and tubulin morphologies. Premeiotic nuclei (devoid of Red1 staining) and multinucleates were not scored in this experiment. Note that in each of these experiments, the wild type control (LY35) and experimental strains (LFT62) each contain a single copy of Zip3-MYC.
(PDF)

Figure S2 Induced Zip1 assembles SC in a SUMO-dependent manner. Chromosome spreads prepared for the *ZIP1* induction experiment (Figure 3) were imaged using the BLAZE OMX Structured Illumination Microscope system (Applied Precision; access kindly provided by Rockefeller's Bio-Imaging Resource Center), in order to determine whether Zip1 assembles normal SC structures in SUMO-diminished nuclei. Each row depicts DAPI-stained chromatin (white, blue in merged image), antibodies to a C terminal fragment of Zip1 (green, 1st, 3rd, 4th, 5th rows) or an N terminal fragment of Zip1 (green, 2nd, 6th, 7th row), and SUMO (red) in either *SMT3+* (top two rows) or *P_SCC1_[SMT3]/smt3Δ* genetic backgrounds. Scale, 1 µm.
(PDF)

Figure S3 *spo11/spo11 P_SCC1_[SMT3]/smt3Δ* sporulating cells form Zip1 polycomplexes devoid of SUMO. (A) Bar graph indicates the percentage of meiotic nuclear spreads that exhibit a Zip1 polycomplex (left) or SUMO polycomplex (right) in *spo11* homozygous diploid cells that are either *SMT3+* (AM1848, shaded box) or *P_SCC1_[SMT3]/smt3Δ* (LFT61, open box). n>50 for each column. (B) Top row shows an example of Zip1 (red) polycomplex with associated SUMO (green) staining in *spo11* homozygous diploid nuclei (DNA in blue). Bottom row shows an example of Zip1 (red) polycomplex devoid of detectable SUMO (green) staining, in *spo11* homozygous diploid nuclei that carry *P_SCC1_[SMT3]/smt3Δ*. Scale, 1 µm. For the data shown in A and B, cells were sporulated for 15 hours.
(PDF)

Figure S4 Ecm11-MYC appears discontinuous in SUMO-diminished pachytene nuclei. (A) Western blot detecting Ecm11-MYC and α-tubulin protein (indicated at right) in lysates from control (K230) and SUMO-diminished (*P_SCC1_[SMT3]/smt3Δ*, K259) cells at 0, 12, 15 18 and 24 hours of sporulation. Bar graph in (B) shows the relative level (normalized between lanes using the tubulin staining) of unSUMOylated Ecm11-MYC (solid), SUMOylated Ecm11-MYC (open), and multi-SUMOylated Ecm11-MYC (boxed) in each lane of the blot shown in (A). (C) Surface-spread meiotic nuclei from K230 (top row) and K259 (bottom two rows) strains that had been sporulated for 24 hours. K230 and K259 strains are homozygous for *ndt80*, thus at 24 hours many surface spread nuclei from each strain are at the pachytene stage of meiosis (which is determined based on the DAPI morphology). DAPI (white, first column) and antibodies to Zip1 (green), Ecm11-MYC (red), SUMO (red) were used to stain nuclei in order to assess SC formation. Scale, 1 µm. (D) displays the percentage of nuclei (n = 30 per column) from these control or SUMO-diminished strains that exhibit either continuous (black), discontinuous (black/white hatched), or dotty Zip1, Ecm11-MYC, or SUMO staining.
(PDF)

Figure S5 Induced Zip1 and Ecm11-MYC distribution are often mutually exclusive in SUMO-diminished strains. Cartoon depicts the Zip1 induction experiment conducted, as described ([46], Figure 3) using (A) K172 and K163 (control homozygous for *ECM11-MYC* or *ecm11(K5R,K101R)-MYC*), and (B) K260 and K262 (SUMO-diminished versions of the above strains). Examples of meiotic surface-spread chromosomes from each strain at three hours post-induction of *ZIP1* expression are shown (genotypes at right indicate strain in each row). Chromosome spreads have been labeled with DAPI (DNA, white), antibodies to Zip1 (green), and Ecm11-MYC (red). Arrows in (B) indicate polycomplex structures. Scale, 1 µm.
(PDF)

Figure S6 Structured illumination reveals parallel tracts of Red1 on paired meiotic chromosomes. Surface spread meiotic chromosomes from AM2712 cells (homozygous for *ECM11-MYC* and *ndt80*) were stained with antibodies to meiotic chromosome axis component Red1 (green), and Ecm11-MYC (red). DAPI (white) stains DNA. Nucleolus is indicated (NOR). Images were taken using Applied Precision's V4 OMX Structured Illumination microscope system (courtesy of Stanford Neuroscience Services, Stanford University). Scale, 1 µm.
(PDF)

Figure S7 Structured illumination versus conventional epifluorescence microscopy. In this comparison, the same slide preparation was imaged and processed using either Applied Precision's Deltavision RT Deconvolution imaging system adapted to an Olympus (IX71) microscope (top row), or Applied Precision's V4 OMX Structured Illumination microscope system (bottom row). In both experiments, DAPI-stained DNA (white, blue in merged image), antibodies against the C terminal 264 amino acids of Zip1 (green) and Ecm11-MYC (red) are imaged. Boxed insets show a zoomed image for the indicated region. Scale, 1 µm.
(PDF)

Figure S8 Ecm11(K5R,K101R)-MYC incorporates into SC structures in *ecm11(K5R,K101R)-MYC/ECM11+* heterozygotes. Meiotic surface spread nuclei from *ECM11-MYC/ECM11+ ndt80/ndt80* (K231, top three rows) and *ecm11(K5R,K101R)-MYC/ECM11+ ndt80/ndt80* (K232, bottom four rows). Chromosome spreads were labeled with DAPI (DNA, blue), antibodies to MYC to label Ecm11-MYC or Ecm11(K5R,K101R) (red) and to Zip1 (green). Scale, 1 µm.
(PDF)

Figure S9 Colocalization of induced Zip1 with Ecm11-MYC and Ecm11(K5R,K101R)-MYC. As shown in the cartoon, Zip1 was induced in K263 and K235 strains (homozygous for *P_GAL1_[ZIP1]*, *ndt80*, and heterozygous for either *ECM11-MYC* or *ecm11(K5R,K101R)-MYC*) at 26 hours of sporulation, and meiotic nuclei were surface spread at 1, 2 or 3 hours after induction. Chromosome spreads were labeled with DAPI (DNA, blue), antibodies to Zip1 (green), and Ecm11-MYC (red). Scale, 1 µm.
(PDF)

Figure S10 Relative abundance of SUMO and Ecm11-MYC correlates with relative Zip1 abundance in SCs containing varying Zip1 levels. Strains with different *ZIP1-YFP* copy numbers (SM224, SM170, SM176, K230, LFT117, LFT119) were used to isolate SCs with varying abundance of Zip1; these SCs were then examined for SUMO and Ecm11-MYC abundance. For strains with either one, two, or four copies of *ZIP1-YFP*, SCs were analyzed for their Zip1-YFP abundance by recording the exposure time that fit a linear range for the image (as in [46]). Optimal exposure times are inversely correlated with the level of Zip1-YFP in each image and thus can be used to assess the relative abundance of the protein within the SC domain. Optimal exposure times were recorded for Zip1-YFP, SUMO and Ecm11-MYC. Note that SUMO and Zip1-YFP were measured in distinct strains from those in which Ecm11-MYC and Zip1-YFP were measured. (A) An example of a surface-spread meiotic nucleus stained with antibodies against Zip1-YFP (green), Ecm11-MYC (red), and DAPI to label DNA. Zoomed insets show examples of the window sizes used to measure optimal exposure time for SC domains. Scale, 1 µm. In (B and C), closed circles represent individual images of SC from either 1, 2, or 4 copy *ZIP1-YFP* strains; each closed circle is plotted according to its optimal Zip1-YFP exposure time (y axis) and its optimal SUMO (B) or Ecm11-MYC (C) exposure time (x axis). For the data in (C), all of the data below the 1-second Zip1-YFP exposure time threshold is also plotted on a separate graph (C, right side) in order to better resolve these data points. Linear regression analysis demonstrated that the positive correlations between SUMO and Zip1-YFP exposure times and Ecm11-MYC and Zip1-YFP exposure times are each extremely significant (r = 0.6141, P<0.0001 in (B); r = 0.4828, P = 0.0004 in (C)).
(PDF)

Table S1 Listed are the strains used in this study. All strains are isogenic with BR1919-8B [53].
(PDF)
