# Supplementary figures and images for: SUMO Localizes to the Central Element of Synaptonemal Complex and Is Required for the Full Synapsis of Meiotic Chromosomes in Budding Yeast
Source: PLoS Genet. 2013 Oct 3;9(10):e1003837. doi: 10.1371/journal.pgen.1003837 (PMC3789832; doi:10.1371/journal.pgen.1003837)

Figure S1

A

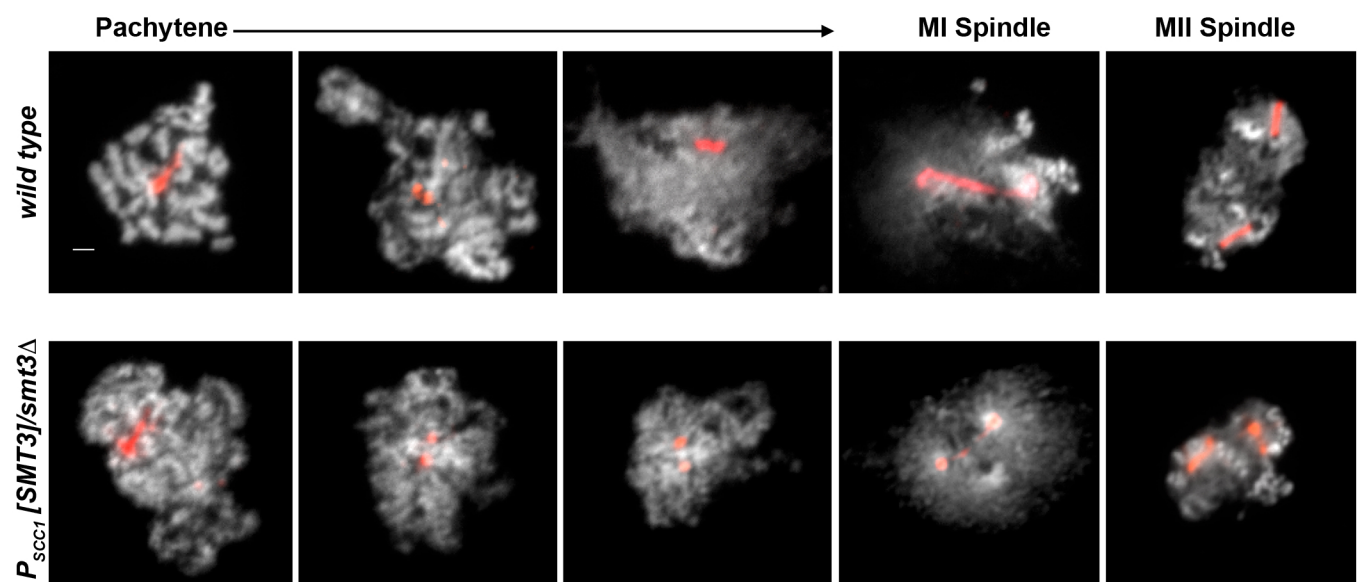

B

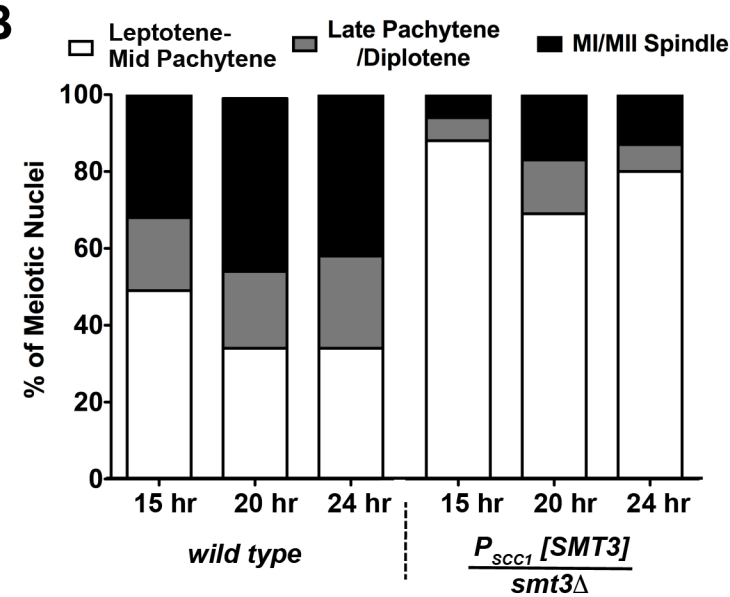

Supplement: Figure S1 — SUMO-diminished strains exhibit delayed meiotic spindle formation. (A) Anti-tubulin and anti-Red1 (not shown) sera were applied to surface-spread nuclei from sporulating cultures of control (LY35) and SUMO-diminished (PSCC1[SMT3]/smt3Δ, LFT62) cells. Examples of the morphology of tubulin structures (red) in meiotic nuclei (DNA, white) at pachytene, diplotene, MI and MII stages from wild type and PSCC1[SMT3]/smt3Δ cells are shown. Note, the microtubules that bridge the duplicated spindle pole bodies in PSCC1[SMT3]/smt3Δ nuclei during post-pachytene stages were frequently diminished. Scale, 1 µm. (B) shows the fraction of surface-spread nuclei that were at early meiotic prophase (leptotene - midpachytene), late pachytene-diplotene, or MI/MII for each time point, assessed based on DAPI, Red1 and tubulin morphologies. Premeiotic nuclei (devoid of Red1 staining) and multinucleates were not scored in this experiment. Note that in each of these experiments, the wild type control (LY35) and experimental strains (LFT62) each contain a single copy of Zip3-MYC. (PDF) [file pgen.1003837.s001.pdf]

Figure S2

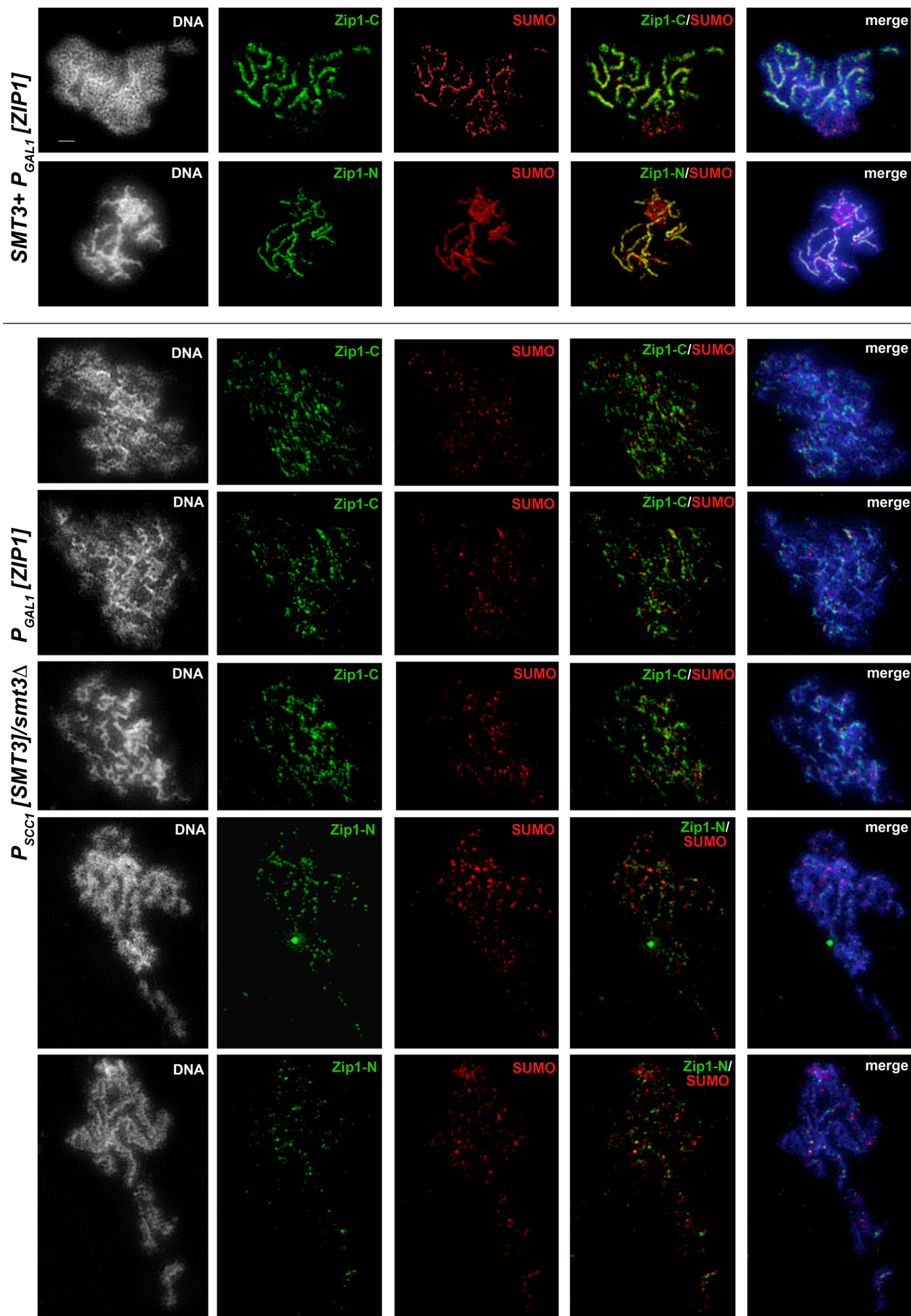

Supplement: Figure S2 — Induced Zip1 assembles SC in a SUMO-dependent manner. Chromosome spreads prepared for the ZIP1 induction experiment (Figure 3) were imaged using the BLAZE OMX Structured Illumination Microscope system (Applied Precision; access kindly provided by Rockefeller's Bio-Imaging Resource Center), in order to determine whether Zip1 assembles normal SC structures in SUMO-diminished nuclei. Each row depicts DAPI-stained chromatin (white, blue in merged image), antibodies to a C terminal fragment of Zip1 (green, 1st, 3rd, 4th, 5th rows) or an N terminal fragment of Zip1 (green, 2nd, 6th, 7th row), and SUMO (red) in either SMT3+ (top two rows) or PSCC1[SMT3]/smt3Δ genetic backgrounds. Scale, 1 µm. (PDF) [file pgen.1003837.s002.pdf]

Figure S3

A

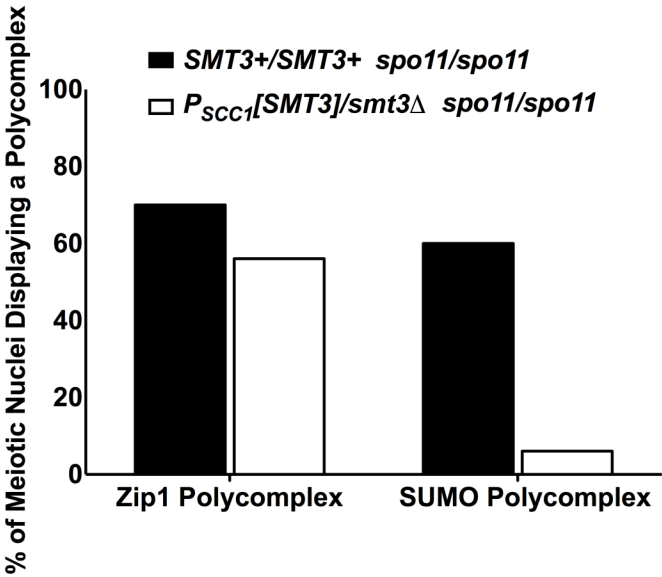

B

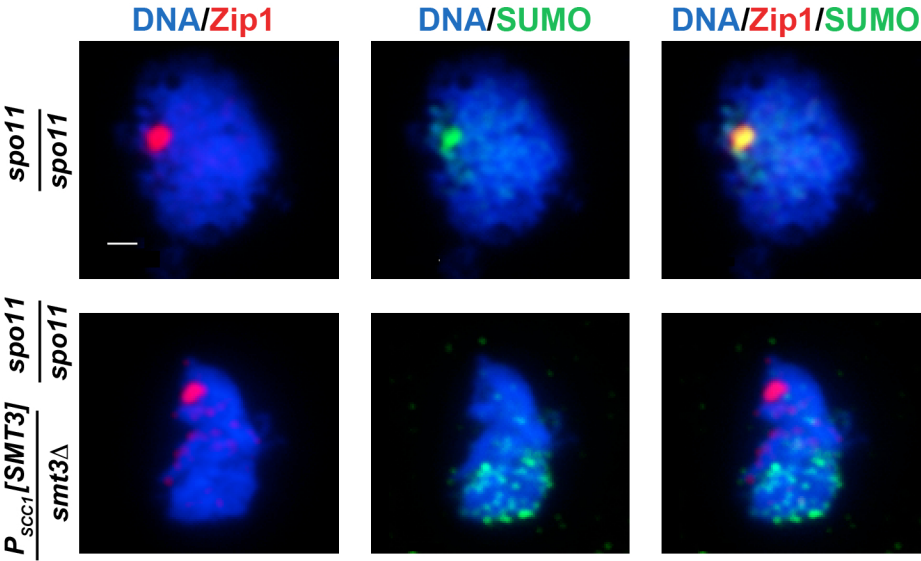

Supplement: Figure S3 — spo11/spo11 PSCC1[SMT3]/smt3Δ sporulating cells form Zip1 polycomplexes devoid of SUMO. (A) Bar graph indicates the percentage of meiotic nuclear spreads that exhibit a Zip1 polycomplex (left) or SUMO polycomplex (right) in spo11 homozygous diploid cells that are either SMT3+ (AM1848, shaded box) or PSCC1[SMT3]/smt3Δ (LFT61, open box). n>50 for each column. (B) Top row shows an example of Zip1 (red) polycomplex with associated SUMO (green) staining in spo11 homozygous diploid nuclei (DNA in blue). Bottom row shows an example of Zip1 (red) polycomplex devoid of detectable SUMO (green) staining, in spo11 homozygous diploid nuclei that carry PSCC1[SMT3]/smt3Δ. Scale, 1 µm. For the data shown in A and B, cells were sporulated for 15 hours. (PDF) [file pgen.1003837.s003.pdf]

Figure S4

A

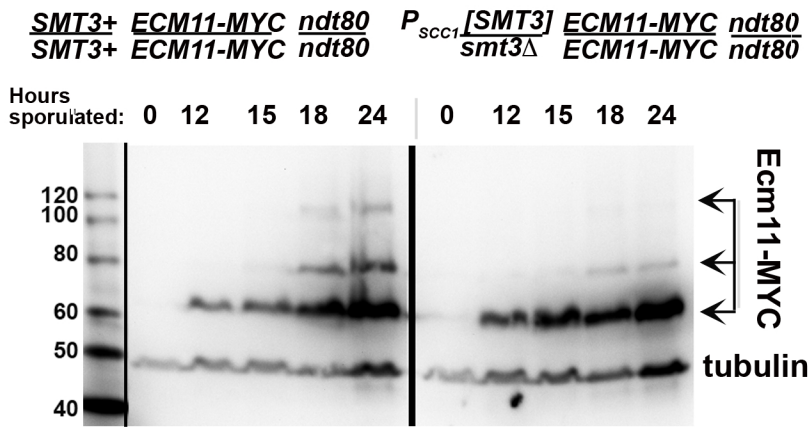

B

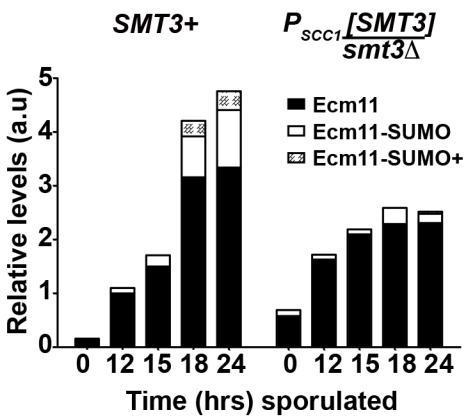

C

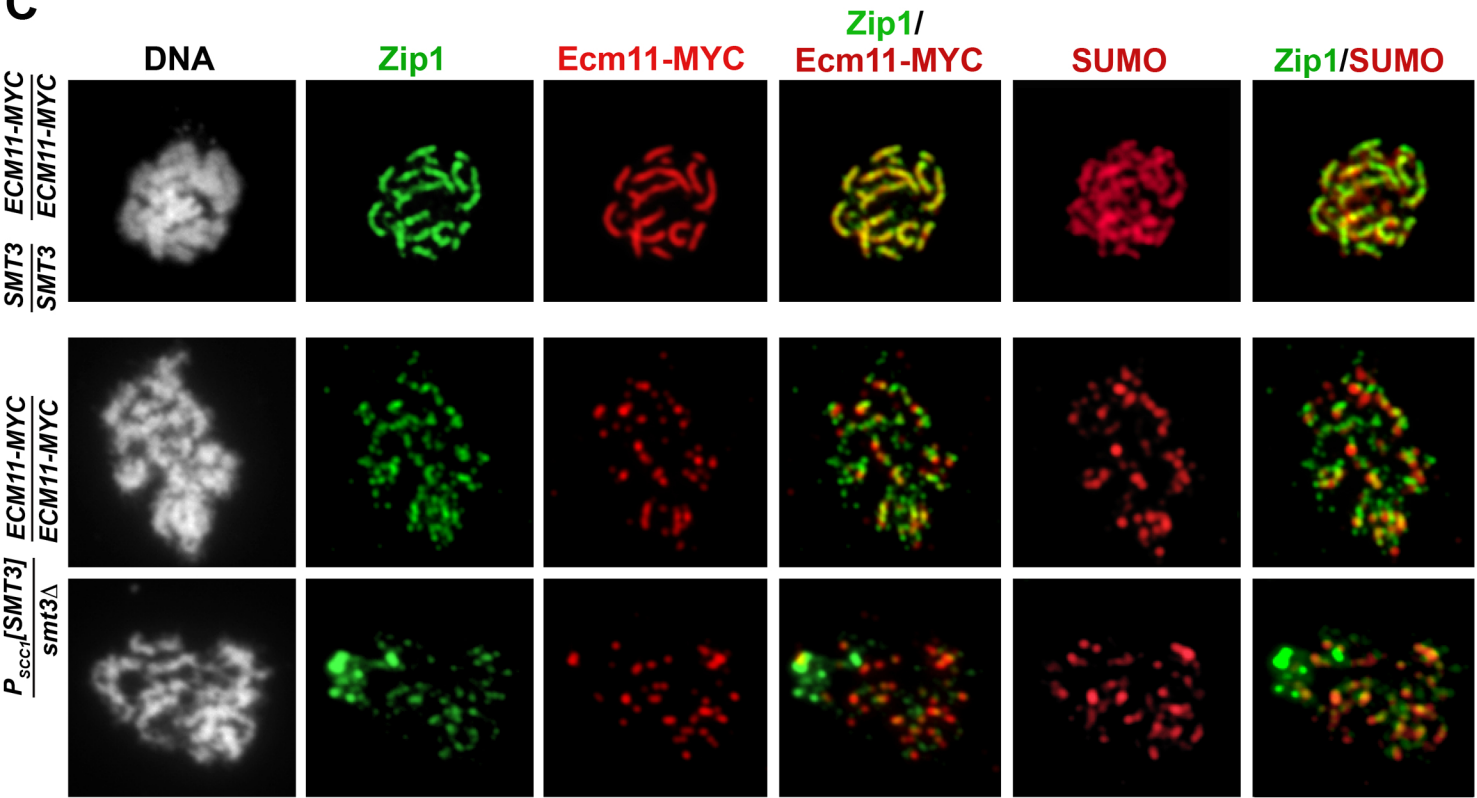

D

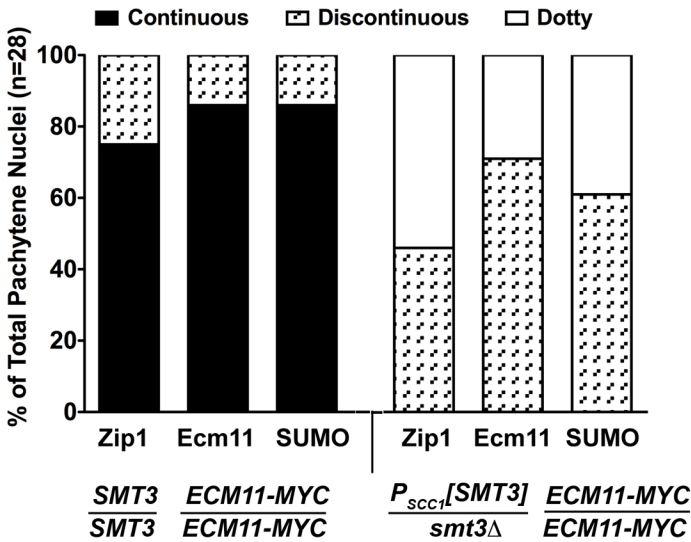

Supplement: Figure S4 — Ecm11-MYC appears discontinuous in SUMO-diminished pachytene nuclei. (A) Western blot detecting Ecm11-MYC and α-tubulin protein (indicated at right) in lysates from control (K230) and SUMO-diminished (PSCC1[SMT3]/smt3Δ, K259) cells at 0, 12, 15 18 and 24 hours of sporulation. Bar graph in (B) shows the relative level (normalized between lanes using the tubulin staining) of unSUMOylated Ecm11-MYC (solid), SUMOylated Ecm11-MYC (open), and multi-SUMOylated Ecm11-MYC (boxed) in each lane of the blot shown in (A). (C) Surface-spread meiotic nuclei from K230 (top row) and K259 (bottom two rows) strains that had been sporulated for 24 hours. K230 and K259 strains are homozygous for ndt80, thus at 24 hours many surface spread nuclei from each strain are at the pachytene stage of meiosis (which is determined based on the DAPI morphology). DAPI (white, first column) and antibodies to Zip1 (green), Ecm11-MYC (red), SUMO (red) were used to stain nuclei in order to assess SC formation. Scale, 1 µm. (D) displays the percentage of nuclei (n = 30 per column) from these control or SUMO-diminished strains that exhibit either continuous (black), discontinuous (black/white hatched), or dotty Zip1, Ecm11-MYC, or SUMO staining. (PDF) [file pgen.1003837.s004.pdf]

0 hr 26hr  
prophase progression  
Zip1 induction  
 $\frac{P_{GAL1} [ZIP1]}{P_{GAL1} [ZIP1]} \frac{GAL4-ER}{GAL4-ER} \frac{ndt80}{ndt80}$

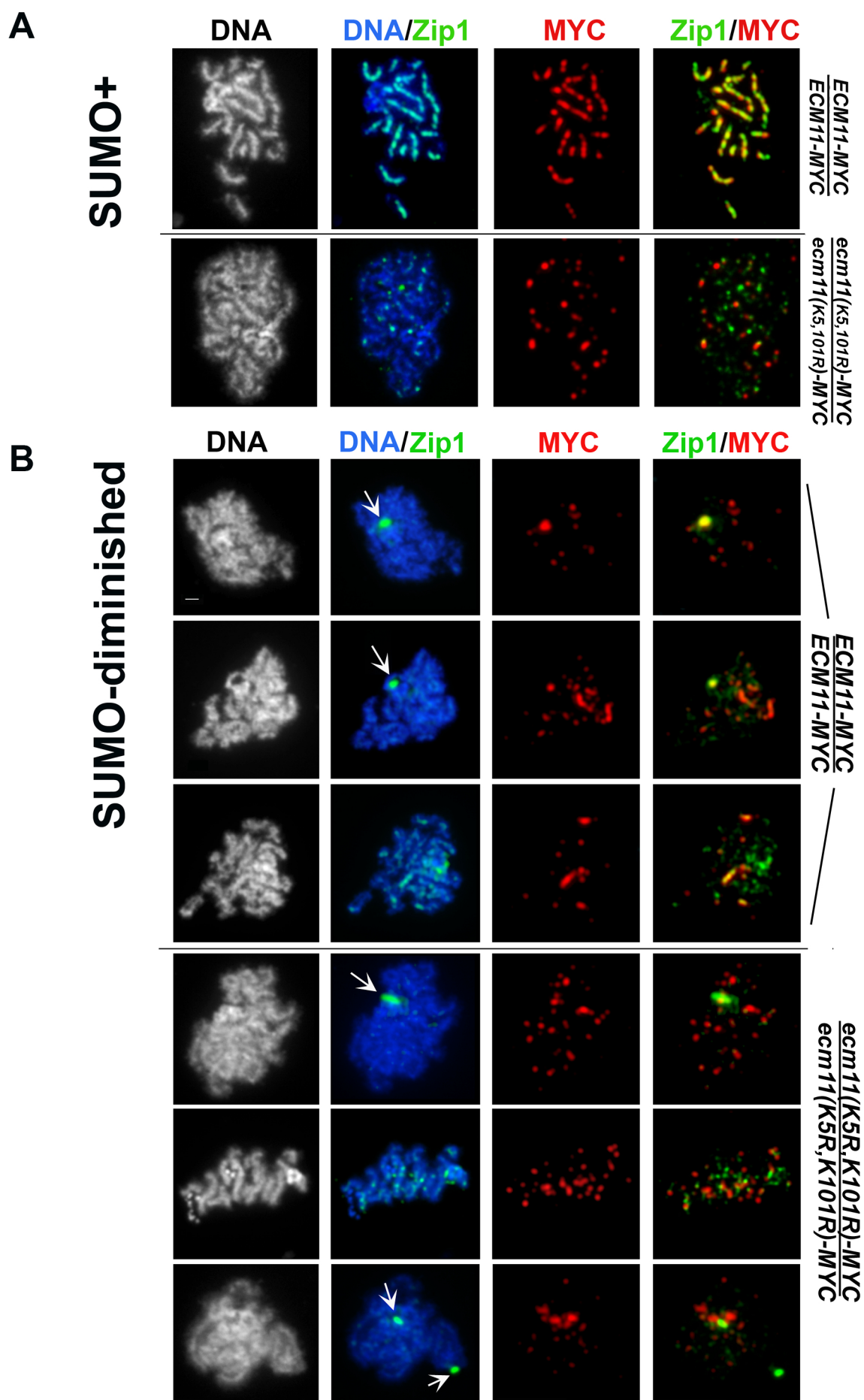

Supplement: Figure S5 — Induced Zip1 and Ecm11-MYC distribution are often mutually exclusive in SUMO-diminished strains. Cartoon depicts the Zip1 induction experiment conducted, as described ([46], Figure 3) using (A) K172 and K163 (control homozygous for ECM11-MYC or ecm11(K5R,K101R)-MYC), and (B) K260 and K262 (SUMO-diminished versions of the above strains). Examples of meiotic surface-spread chromosomes from each strain at three hours post-induction of ZIP1 expression are shown (genotypes at right indicate strain in each row). Chromosome spreads have been labeled with DAPI (DNA, white), antibodies to Zip1 (green), and Ecm11-MYC (red). Arrows in (B) indicate polycomplex structures. Scale, 1 µm. (PDF) [file pgen.1003837.s005.pdf]

Figure S6

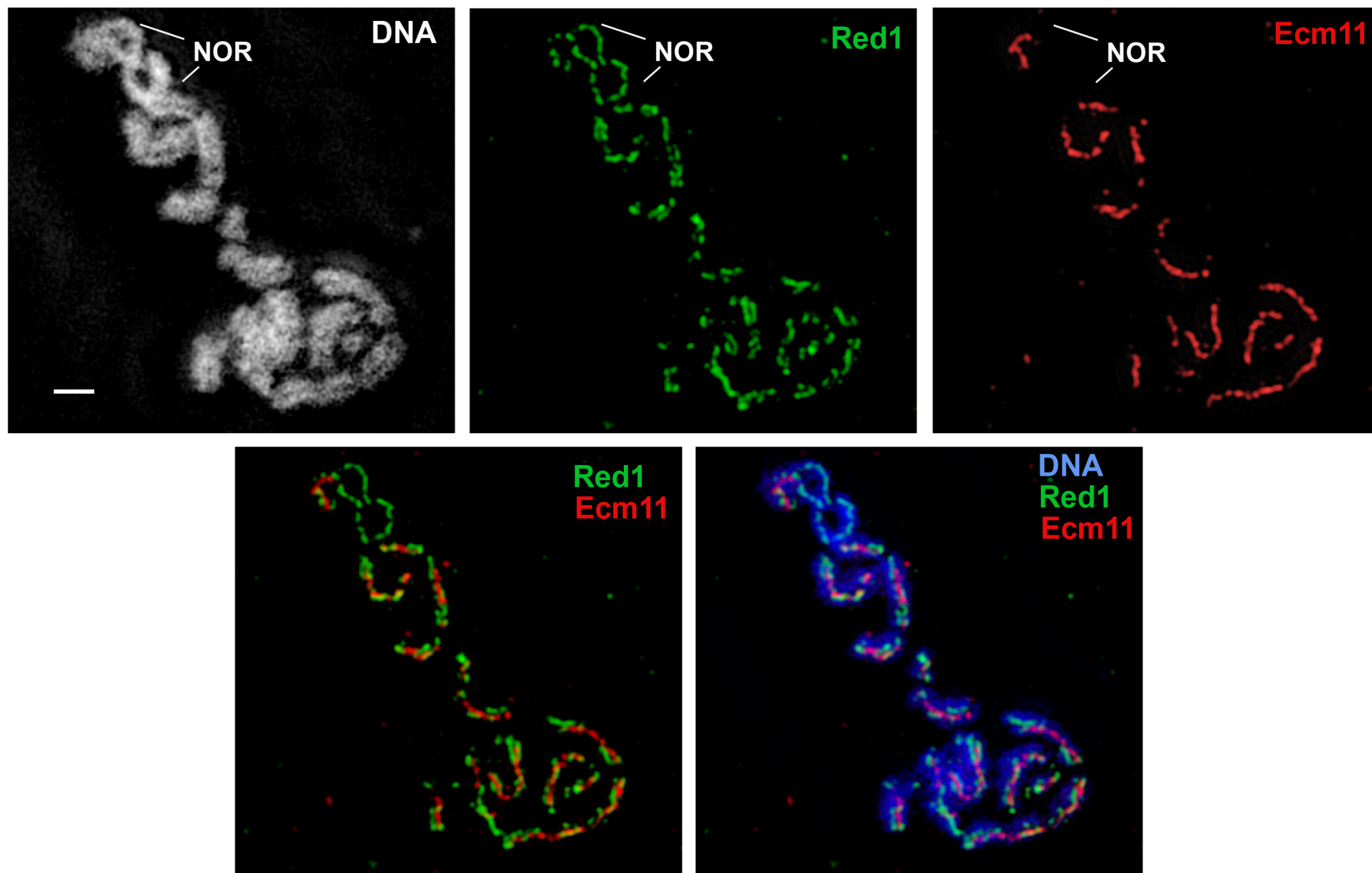

Supplement: Figure S6 — Structured illumination reveals parallel tracts of Red1 on paired meiotic chromosomes. Surface spread meiotic chromosomes from AM2712 cells (homozygous for ECM11-MYC and ndt80) were stained with antibodies to meiotic chromosome axis component Red1 (green), and Ecm11-MYC (red). DAPI (white) stains DNA. Nucleolus is indicated (NOR). Images were taken using Applied Precision's V4 OMX Structured Illumination microscope system (courtesy of Stanford Neuroscience Services, Stanford University). Scale, 1 µm. (PDF) [file pgen.1003837.s006.pdf]

Figure S7

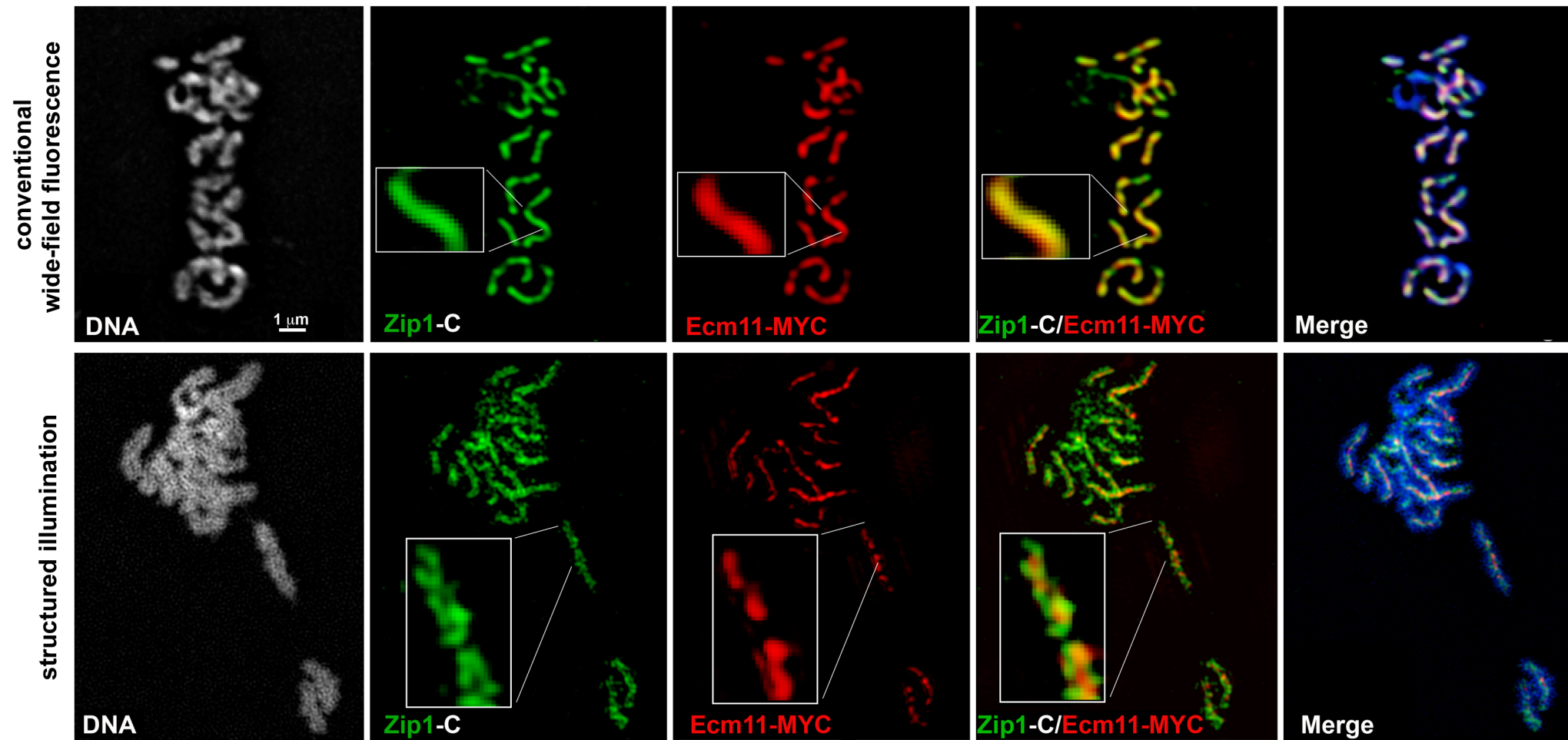

Supplement: Figure S7 — Structured illumination versus conventional epifluorescence microscopy. In this comparison, the same slide preparation was imaged and processed using either Applied Precision's Deltavision RT Deconvolution imaging system adapted to an Olympus (IX71) microscope (top row), or Applied Precision's V4 OMX Structured Illumination microscope system (bottom row). In both experiments, DAPI-stained DNA (white, blue in merged image), antibodies against the C terminal 264 amino acids of Zip1 (green) and Ecm11-MYC (red) are imaged. Boxed insets show a zoomed image for the indicated region. Scale, 1 µm. (PDF) [file pgen.1003837.s007.pdf]

Figure S8

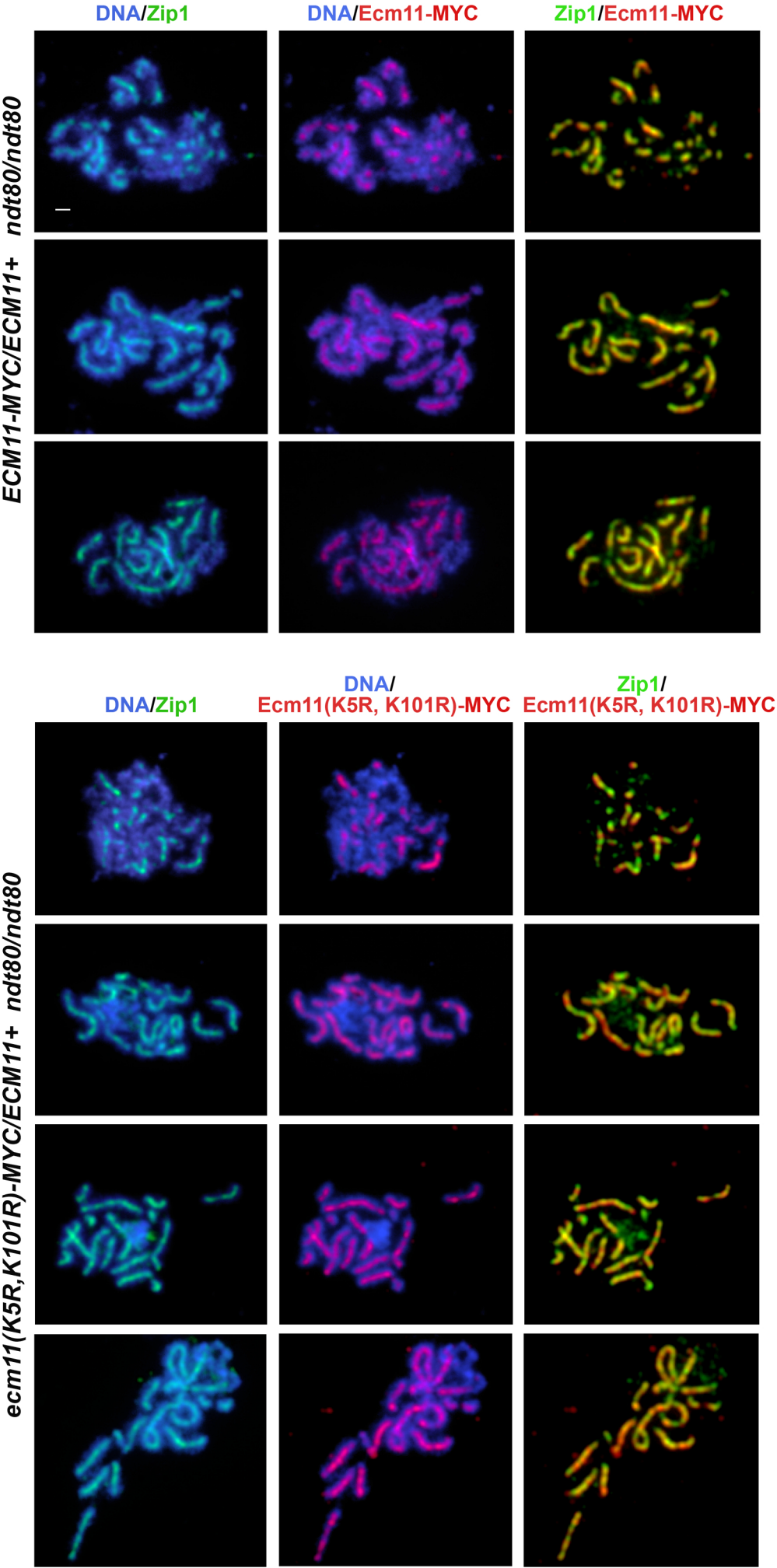

Supplement: Figure S8 — Ecm11(K5R,K101R)-MYC incorporates into SC structures in ecm11(K5R,K101R)-MYC/ECM11+ heterozygotes. Meiotic surface spread nuclei from ECM11-MYC/ECM11+ ndt80/ndt80 (K231, top three rows) and ecm11(K5R,K101R)-MYC/ECM11+ ndt80/ndt80 (K232, bottom four rows). Chromosome spreads were labeled with DAPI (DNA, blue), antibodies to MYC to label Ecm11-MYC or Ecm11(K5R,K101R) (red) and to Zip1 (green). Scale, 1 µm. (PDF) [file pgen.1003837.s008.pdf]

Figure S9

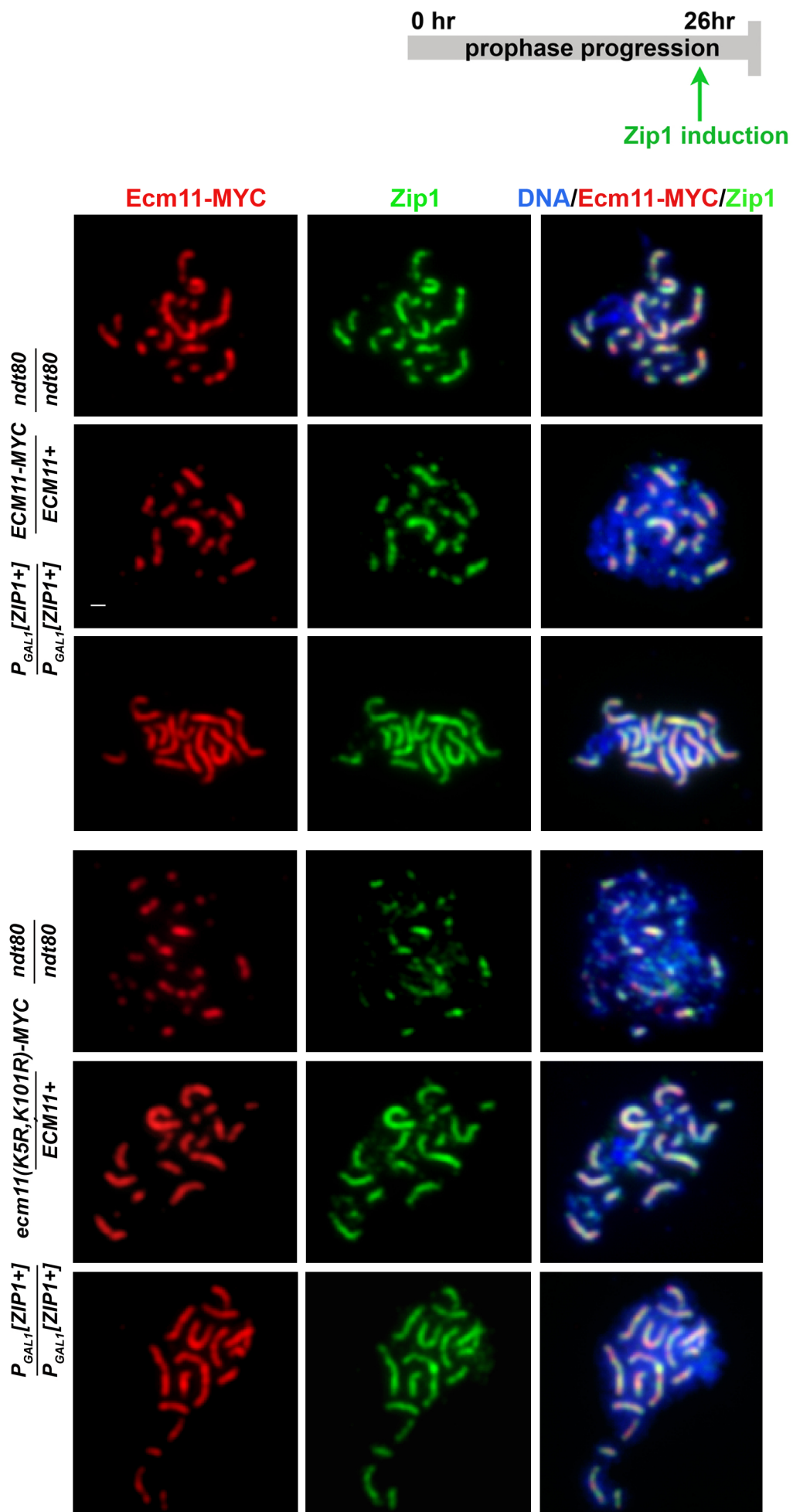

Supplement: Figure S9 — Colocalization of induced Zip1 with Ecm11-MYC and Ecm11(K5R,K101R)-MYC. As shown in the cartoon, Zip1 was induced in K263 and K235 strains (homozygous for PGAL1[ZIP1], ndt80, and heterozygous for either ECM11-MYC or ecm11(K5R,K101R)-MYC) at 26 hours of sporulation, and meiotic nuclei were surface spread at 1, 2 or 3 hours after induction. Chromosome spreads were labeled with DAPI (DNA, blue), antibodies to Zip1 (green), and Ecm11-MYC (red). Scale, 1 µm. (PDF) [file pgen.1003837.s009.pdf]

Figure S10

A

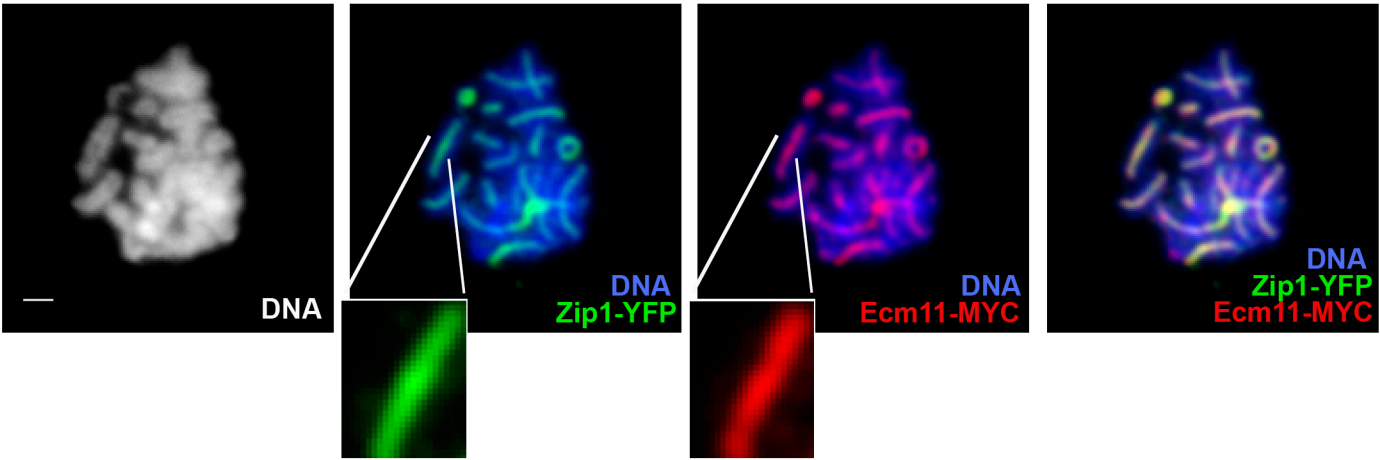

B

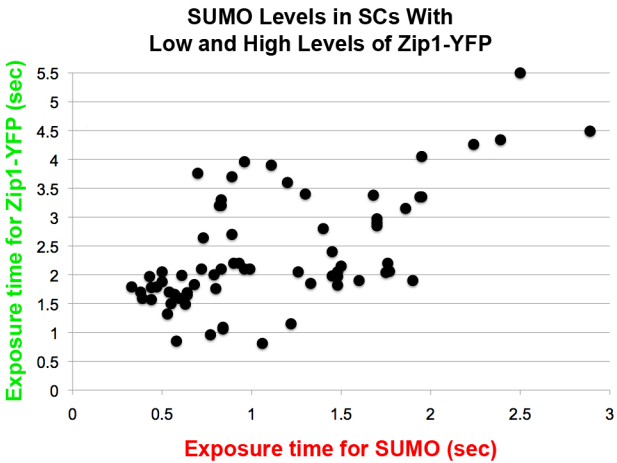

C

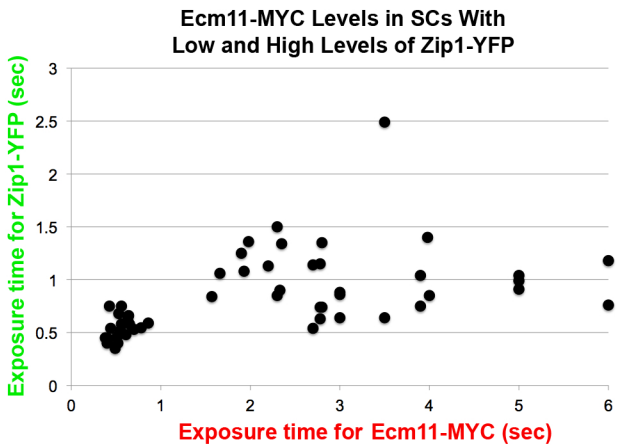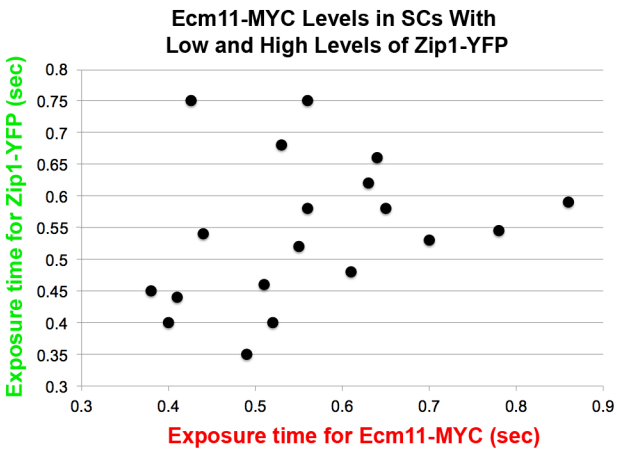

Supplement: Figure S10 — Relative abundance of SUMO and Ecm11-MYC correlates with relative Zip1 abundance in SCs containing varying Zip1 levels. Strains with different ZIP1-YFP copy numbers (SM224, SM170, SM176, K230, LFT117, LFT119) were used to isolate SCs with varying abundance of Zip1; these SCs were then examined for SUMO and Ecm11-MYC abundance. For strains with either one, two, or four copies of ZIP1-YFP, SCs were analyzed for their Zip1-YFP abundance by recording the exposure time that fit a linear range for the image (as in [46]). Optimal exposure times are inversely correlated with the level of Zip1-YFP in each image and thus can be used to assess the relative abundance of the protein within the SC domain. Optimal exposure times were recorded for Zip1-YFP, SUMO and Ecm11-MYC. Note that SUMO and Zip1-YFP were measured in distinct strains from those in which Ecm11-MYC and Zip1-YFP were measured. (A) An example of a surface-spread meiotic nucleus stained with antibodies against Zip1-YFP (green), Ecm11-MYC (red), and DAPI to label DNA. Zoomed insets show examples of the window sizes used to measure optimal exposure time for SC domains. Scale, 1 µm. In (B and C), closed circles represent individual images of SC from either 1, 2, or 4 copy ZIP1-YFP strains; each closed circle is plotted according to its optimal Zip1-YFP exposure time (y axis) and its optimal SUMO (B) or Ecm11-MYC (C) exposure time (x axis). For the data in (C), all of the data below the 1-second Zip1-YFP exposure time threshold is also plotted on a separate graph (C, right side) in order to better resolve these data points. Linear regression analysis demonstrated that the positive correlations between SUMO and Zip1-YFP exposure times and Ecm11-MYC and Zip1-YFP exposure times are each extremely significant (r = 0.6141, P<0.0001 in (B); r = 0.4828, P = 0.0004 in (C)). (PDF) [file pgen.1003837.s010.pdf]
