# Supplementary material for: SUMO Localizes to the Central Element of Synaptonemal Complex and Is Required for the Full Synapsis of Meiotic Chromosomes in Budding Yeast
Source: PLoS Genet. 2013 Oct 3;9(10):e1003837. doi: 10.1371/journal.pgen.1003837 (PMC3789832; doi:10.1371/journal.pgen.1003837)
Supplement: Table S1 — Listed are the strains used in this study. All strains are isogenic with BR1919-8B [53]. (PDF) [file pgen.1003837.s011.pdf]

Table S1. Strains used in this study

NAME	GENOTYPE
**PM89**	*MATα leu2-3,112 his4-260,519 lys2ΔNhe thr1-4 ade2-1 ura3-1 trp1-289*<br>*MATa leu2-3,112 his4-260,519 lys2ΔNhe thr1-4 ade2-1 ura3-1 trp1-289*
**AM447**	PM89 *LYS2 ndt80Δ::URA3*<br>*LYS2 ndt80Δ::URA3*
**LFT36**	PM89 *smt3Δ::hphMX4*<br>*SMT3*
**LFT46**	LFT36 *kanMX4-*P_{SCC1}*-SMT3*<br>*smt3Δ::hphMX4*
**LY35**	PM89 *Zip3-MYC*<br>*ZIP3*
**LFT62**	LY35 *kanMX4-*P_{SCC1}*-SMT3*<br>*smt3Δ::hphMX4*
**AM2712**	PM89 *ECM11-13MYC::kanMX4 ndt80Δ::URA3*<br>*ECM11-13MYC::kanMX4 ndt80Δ::URA3*
**LFT51**	PM89 *TRP1-*P_{GAL1}*-ZIP1 ura3::*P_{GPD1}*-GAL4(848).ER::URA3 ndt80Δ::LEU2*<br>*TRP1-*P_{GAL1}*-ZIP1 URA3 ndt80Δ::LEU2*
**LFT65**	LFT51 *kanMX4-*P_{SCC1}*-SMT3*<br>*smt3Δ::hphMX4*
**AM2905**	PM89 *TRP1-*P_{GAL1}*-V5-SMT3 TRP1-*P_{GAL1}*-ZIP1-GFP ura3::*P_{GPD1}*-GAL4(848).ER::URA3*<br>*SMT3 ZIP1 ura3::*P_{GPD1}*-GAL4(848).ER::URA3*<br>*ndt80Δ::LEU2*<br>*ndt80Δ::LEU2*
**AM2865**	PM89 *TRP1-*P_{GAL1}*-ECM11-13MYC::kanMX4 TRP1-*P_{GAL1}*-ZIP1-GFP*<br>*ECM11 ZIP1*<br>*ura3::*P_{GPD1}*-GAL4(848).ER::URA3 ndt80Δ::LEU2*<br>*ura3::*P_{GPD1}*-GAL4(848).ER::URA3 ndt80Δ::LEU2*
**AM2910**	PM89 *TRP1-*P_{GAL1}*-ecm11(K5R, K101R)-13MYC::kanMX4 TRP1-*P_{GAL1}*-ZIP1-GFP*<br>*ECM11 ZIP1*<br>*ura3::*P_{GPD1}*-GAL4(848).ER::URA3 ndt80Δ::LEU2*<br>*ura3::*P_{GPD1}*-GAL4(848).ER::URA3 ndt80Δ::LEU2*
**SB14**	PM89 *ZIP1-GFP ndt80Δ::LEU2*<br>*ZIP1-GFP ndt80Δ::LEU2*
**LFT37**	SB14 *smt3Δ::hphMX4*<br>*SMT3*
**LFT47**	SB14 *kanMX4-*P_{SCC1}*-SMT3*<br>*smt3Δ::hphMX4*
**LFT80**	PM89 *pch2 Δ::hphMX4*<br>*pch2 Δ::hphMX4*
**LFT85**	LFT80 *kanMX4-*P_{SCC1}*-SMT3*<br>*smt3Δ::natMX4*
**AM1848**	PM89 *LYS2 spo11Δ::ADE2 ndt80Δ::LEU2*<br>*LYS2 spo11Δ::ADE2 ndt80Δ::LEU2*
**LFT61**	AM1848 *kanMX4-*P_{SCC1}*-SMT3*<br>*smt3Δ::hphMX4*
**K231**	PM89 *ECM11-13MYC::kanMX4 ndt80Δ::LEU2*<br>*ECM11 ndt80Δ::LEU2*
**K232**	PM89 *ecm11(K5R, K101R)-13MYC::kanMX4 ura3::*P_{GPD1}*-GAL4(848).ER::URA3 ndt80Δ::LEU2*<br>*ECM11 ura3-1 ndt80Δ::LEU2*

**K172**	LFT51 ECM11-13MYC::kanMX4<br>ECM11-13MYC::kanMX4
**K163**	LFT51 ecm11(K5R, K101R)-13MYC::kanMX4<br>ecm11(K5R, K101R)-13MYC::kanMX4
**K263**	LFT51 ECM11-13MYC::kanMX4 LYS2<br>ECM11 LYS2
**K235**	LFT51 ecm11(K5R, K101R)-13MYC::kanMX4<br>ECM11
**K260**	K172 kanMX4-P_{SCC1}-SMT3<br>smt3Δ::hphMX4
**K262**	K163 kanMX4-P_{SCC1}-SMT3<br>smt3Δ::hphMX4
**K230**	K172 ndt80Δ::LEU2<br>ndt80Δ::LEU2
**K259**	K230 kanMX4-P_{SCC1}-SMT3<br>smt3Δ::hphMX4
**K189**	PM89 his4-260,519 CEN3 RAD18 TetO:THR1(@215,000 of IV) LacO-CEN4-LEU2<br>HIS4 HYG@CEN3 ADE2@RAD18 CEN4
**K188**	K189 kanMX4-P_{SCC1}-SMT3<br>kanMX4-P_{SCC1}-SMT3
**K197**	K189 smt3Δ::natMX4<br>SMT3
**K196**	K189 kanMX4-P_{SCC1}-SMT3<br>smt3Δ::natMX4
**K167**	K189 pch2::TRP1<br>pch2::TRP1
**K191**	K167 kanMX4-P_{SCC1}-SMT3<br>kanMX4-P_{SCC1}-SMT3
**K325**	K167 smt3Δ::natMX4<br>SMT3
**K198**	K189 pch2::TRP1 kanMX4-P_{SCC1}-SMT3<br>pch2::TRP1 smt3Δ::natMX4
**K223**	K167 PCH2 kanMX4-P_{SCC1}-SMT3<br>PCH2 smt3Δ::natMX4
**AM2892**	PM89 ECM11-13MYC::kanMX4 ndt80Δ::LEU2<br>ECM11+ ndt80Δ::LEU2
**SM170**	PM89 TRP1-P_{GAL1}-ZIP1 ura3::P_{GPD1}-GAL4(848).ER::URA3 ndt80Δ::LEU2<br>ZIP1-YFP URA3 ndt80Δ::LEU2
**SM176**	PM89 ZIP1-YFP ndt80Δ::LEU2<br>ZIP1-YFP ndt80Δ::LEU2
**SM224**	PM89 ZIP1-YFP 2x[ZIP1-YFP]@LEU2 ndt80Δ::HYG<br>ZIP1-YFP leu2 ndt80Δ::HYG
**LFT117**	PM89 ZIP1-YFP ECM11-13MYC::kanMX4 ndt80Δ::LEU2<br>TRP1-P_{GAL1}-ZIP1 ECM11-13MYC::kanMX4 ndt80Δ::LEU2
**LFT119**	PM89 ZIP1-YFP 2x[ZIP1-YFP]@LEU2 ECM11-13MYC::kanMX4 ndt80Δ::HYG<br>ZIP1-YFP leu2 ECM11-13MYC::kanMX4 ndt80Δ::HYG
